# H-shift and cyclization reactions in unsaturated alkylperoxy radicals near room temperature: propagating or terminating autoxidation?†

**DOI:** 10.1039/d4cp02718c

**Published:** 2024-09-16

**Authors:** Barbara Nozière, Luc Vereecken

**Affiliations:** a Department of Chemistry, Royal Institute of Technology, KTH 11428 Stockholm Sweden noziere@kth.se; b Institute of Climate and Energy Systems, ICE-3: Troposphere Forschungszentrum Jülich GmbH Jülich Germany L.Vereecken@fz-juelich.de

## Abstract

The autoxidation of alkylperoxy radicals (RO_2_, where R is organic) is an important degradation pathway for organic compounds in a wide range of chemical systems including Earth's atmosphere. It is thought to proceed by internal H-shift reactions and, for unsaturated radicals, cyclization. However, experimental data on specific reactions steps for unsaturated RO_2_ is scarce. This work investigates the unimolecular reactions of 1-butenyl-O_2_, 1-pentenyl-O_2_, 1-hexenyl-O_2_, and 2-methyl-2-pentenyl-O_2_ radicals near room temperature (302 ± 3 K) experimentally, by monitoring the radicals directly, and theoretically. The experimental rate coefficients are in good agreement with those determined with high-level quantum calculations, confirming that cyclization can be competitive with H-shift in some cases. However, the products observed experimentally with two different mass spectrometers suggest that all the peroxy radicals studied lead to fast decomposition (*k* > 1 s^−1^) after the isomerization step. While the mechanisms for these decompositions could not be fully elucidated theoretically, they question whether these channels contribute to propagation or to termination of the autoxidation chains.

## Abstract 2

Experimental and theoretical rate coefficients for unimolecular reactions of gas-phase unsaturated organic peroxy radicals (RO_2_) are in excellent agreement. But the observed products question their contributions to autoxidation in Earth’s atmosphere.

## Graphical abstract

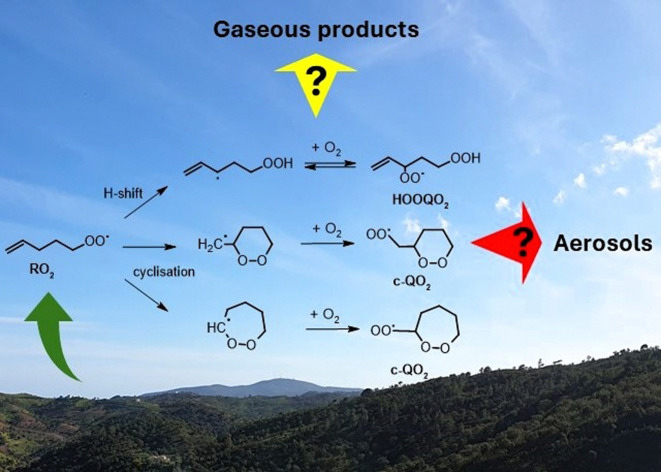

## Introduction

**Scheme 1:**
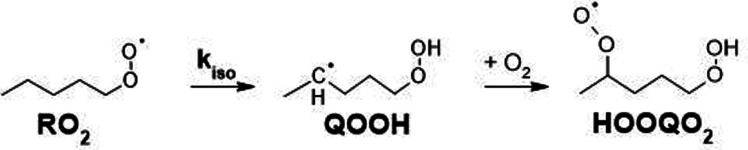
H-shift reaction illustrated for 1-pentyl-O_2_ radical.

The autoxidation of alkylperoxy radicals (RO_2_, where R is organic) is a major degradation pathway for organic compounds in the dark and under oxygenated conditions. These processes are thus important in a wide range of chemical systems including natural ones (lipid peroxidation in living organisms)^1^ and industrial applications (chemical industry, food industry…). After being overlooked for decades in the chemistry of Earth's atmosphere, these reactions are now also considered as important sources of semi- and non-volatile compounds and, ultimately, new aerosol particles.^2^ However, in spite of numerous investigations, these processes are still not completely elucidated and even less quantified. The main reaction steps considered in autoxidation are H-shift reactions (Scheme 1), in which a RO_2_ produces a “HOOQ” alkyl radical and, after further addition of O_2_, a “HOOQO_2_” peroxy radical.

**Scheme 2:**
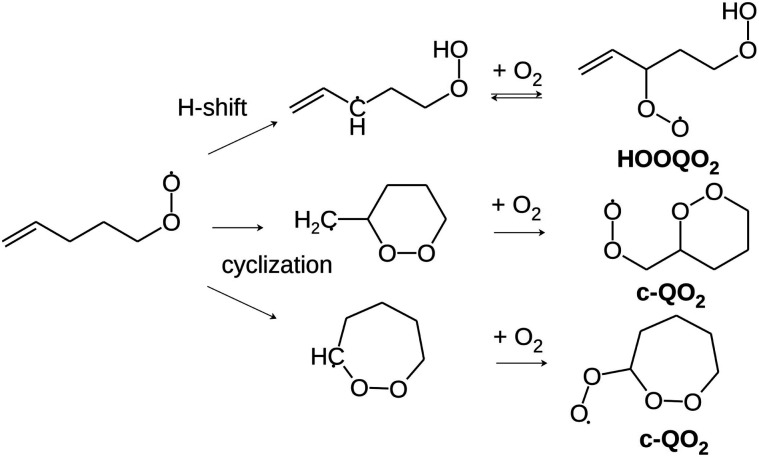
Illustration of the cyclization pathways competing with the allylic H-shift pathway in 1-pentenyl-O_2_. For clarity, the slower migrations of aliphatic H-atoms are not shown. O_2_ addition on an allyl radical is reversible, which affects the subsequent chemistry.

These H-shift reactions have been the subject of numerous experimental and theoretical studies, especially for alkyl and oxygenated RO_2_.^3^ Structure activity relationships (SAR) have been proposed to predict the rate coefficients for the H-shift reactions of RO_2_ with a wide range of molecular structures.^4–6^ This included unsaturated RO_2_ that are especially relevant for the atmospheric oxidation of unsaturated organic compounds such as the ubiquitous isoprene and terpenes, abundantly emitted into the atmosphere from vegetation. For unsaturated RO_2_, theoretical studies have shown that migration of allylic H-atoms can be fast due to the resonance stabilization of the QOOH products, and contributes strongly to autoxidation.^6,7^ However, the vinyl-H-atoms do not migrate and can not help to oxidize double- bonded carbons. Thus, H-migrations alone can not account for the oxidation of (nearly) all carbons and for the O:C ratios of up to 2 : 1 reported by mass-spectrometric investigations even for unsaturated VOCs.^8–11^ Theoretical investigations have shown that, for unsaturated RO_2_, cyclization (Scheme 2), producing a peroxy radical carrying a peroxide heterocycle (thereafter referred to as “c-QO_2_”), could be competitive with H-migration, and provide a pathway for activating double-bonded carbons for autoxidation.^12–14^ A recent SAR was established to predict the rate coefficients for these cyclization reactions.^14^ However, experimental data that would validate this new SAR is scarce, especially for individual reactions and specific RO_2_. This is because, until now, experimental investigations of the gas-phase autoxidation of unsaturated compounds were mostly performed on complex systems, in which a single precursor (isoprene, terpene) produces many different RO_2_ simultaneously.^15^ The numerous reactions taking place simultaneously in such systems make the kinetic analysis of individual reaction steps difficult. This is even more the case with unsaturated RO_2_ than with aliphatic systems because *e.g.* the reversibility of the O_2_ addition on allylic radicals makes the mechanisms even more complex.^15–18^ To provide rate coefficients for the H-shift and cyclization reactions of individual unsaturated RO_2_ and compare with the recent SAR predictions, the present work proposes an experimental and theoretical study of the reactions of 1-butenyl-O_2_, 1-pentenyl-O_2_, 1-hexenyl-O_2_, and 2-methyl-2-pentenyl-O_2_ radicals. In addition to monitoring directly the radicals for the kinetic analysis, reaction products were also analyzed to determine the fate of the HOOQO_2_ and c-QO_2_ radicals and propose some mechanisms for the reactions.

## Experimental methods

### Flow reactor experiments

All the experiments were performed in a vertical Quartz reactor described previously^19^ (internal diameter *d* = 5 cm, total length *L* = 120 cm only the lower half, ∼60 cm, being used in this study). The bath gas was synthetic air with mass flows between 1.00 and 3.75 sLm operated in continuous flow and at a total pressure *P* = 0.9 bar. Under these conditions the flow through the reactor was in the laminar regime. The reactions were performed at 300 ± 3 K. The radical iodinated or brominated precursors were transferred to the gas phase by bubbling a small flow of N_2_ through the pure liquids. These small flows were introduced in the reactor at mid length. The radicals were produced by photolyzing the iodinated or brominated precursors over small irradiation “windows”, using 4 narrow-band UV-C lamps (Phillips TUV 36W SLV/6) emitting at *λ* = 254 nm, the remainder of the reactor being kept in the dark with aluminum foil. For instance, for methyl peroxy:

1 CH_3_I + *hν* → CH_3_ + I

2 CH_3_ + O_2_ + M → CH_3_O_2_ + M

The length of these irradiation windows was chosen to maximize the production of radicals and corresponded to a residence time of ∼8 s, for the iodinated precursors and 15 to 20 s for the brominated ones. Below these irradiation windows various lengths of the reactor, between 1 and 10 cm, were kept in the dark to let the radicals react over reaction times of 0 to 10 s. At the bottom of the reaction section a fraction of the total flow was sampled through an inlet for analysis, either with a chemical-ionisation quadrupole mass spectrometer (CIMS), for RO_2_ monitoring and kinetic analysis, or with a proton-transfer-reaction time-of-flight mass-spectrometer (PTR-TOF-MS), for product analysis. A complete list of the experiments performed and of the experimental conditions is given in Table S2 (ESI†).

### Radical detection with CIMS

All the organic peroxy radicals present in the reaction systems were detected with the CIMS instrument, using proton transfer with the parent ions H_3_O^+^ and its water clusters, (H_2_O)_*n*_H^+^ (with *n* = 2–3), following the reaction:^3,20–22^

3 RO_2_ + (H_2_O)_*n*_H^+^ → RO_2_(H_2_O)_*n*−1_H^+^ + H_2_O.

A radical of molecular mass *M* was thus detected by its ion products at *m*/*z* = M + 1, M + 19, M + 37, M + 55, *etc.* As in our previous works, to distinguish the signals specifically due to the RO_2_ from potential interferences from other compounds, excess NO was periodically added to the reactor, to consume all the RO_2_ present. The residual signals obtained by subtracting those obtained in the presence of NO from those obtained in the absence of NO thus corresponded solely to the RO_2_. The concentration of radicals produced in the reactor were estimated from the vapor pressure of their iodinated (or brominated) precursors, from the photolysis rates for the precursors, determined experimentally in the reactor, and by applying the kinetic model detailed in Section S4 of the ESI.† The main uncertainties on these concentrations were due to those on the vapor pressure of the precursors, which are not well established in the literature. Additional uncertainties in the reaction rate coefficients obtained from the kinetic analysis resulted from uncertainties of ∼0.5 s on the reaction times.

### Detection of the stable products

The stable products present in the reaction mixtures were analyzed in real time both with the CIMS and with PTR-TOF-MS using a FUSION PTR-TOF 10 k (Ionicon Analytik Gmbh, Innsbruck, Austria). The PTR-TOF-MS instrument^23^ includes an orthogonal acceleration reflectron time-of-flight, providing a mass resolution of about 7000. The drift tube, where the proton-analyte reactions take place, was equipped with direct current (DC) and radiofrequency (RF) electric fields. A source generated H_3_O^+^ in the sampled flow by proton-transfer:

4 H_3_O^+^ + A → AH^+^ + H_2_O

The drift tube was operated around 3.8 mbar and with a voltage of 250 V, corresponding to a reduced electric field of *E*/*N* = ∼40 Td. The data were analyzed with the PTR-MS Viewer software V3.4.3.12 (Ionicon Analytik Gmbh, Innsbruck, Austria) and in-house Julia scripts.

### Chemicals

4-Iodobut-1-ene (CAS 7766-51-0), 99%, Aldrich; 5-iodopent-1-ene (CAS 7766-48-5), 97%, Acros; 6-iodo-1-hexene (CAS 18922-04-8), 98%, Acros; 5-Bromo-2-methyl-2-pentene (CAS 2270-59-9), 98%, Sigma. Gases: Synthetic air, 5.0, Linde Gas; NO, 200 ppmV in N_2_, Air Liquide.

## Theoretical method

The rate predictions are based on CCSD(T)/aug-cc-pVTZ//M06-2X-D3/aug-cc-pVTZ characterization of the potential energy surfaces, including all conformers of reactants and transition states.^24^ All pathways are validated using intrinsic reaction coordinate (IRC) calculations. These calculations were performed using Gaussian-16.^25^ Subsequently, the temperature-dependent high-pressure rate is determined using multi-conformer transition state theory (MC-TST), incorporating all conformers in the kinetic analysis.^26^ The expected rate uncertainty is a factor 2 to 3. For some exploratory calculations, data at the M06-2X-D3/aug-cc-pVTZ level of theory was used (see ESI†).

## Results and discussion

### Experimental determination of the rate coefficients for the unimolecular reactions

**Fig. 1:**
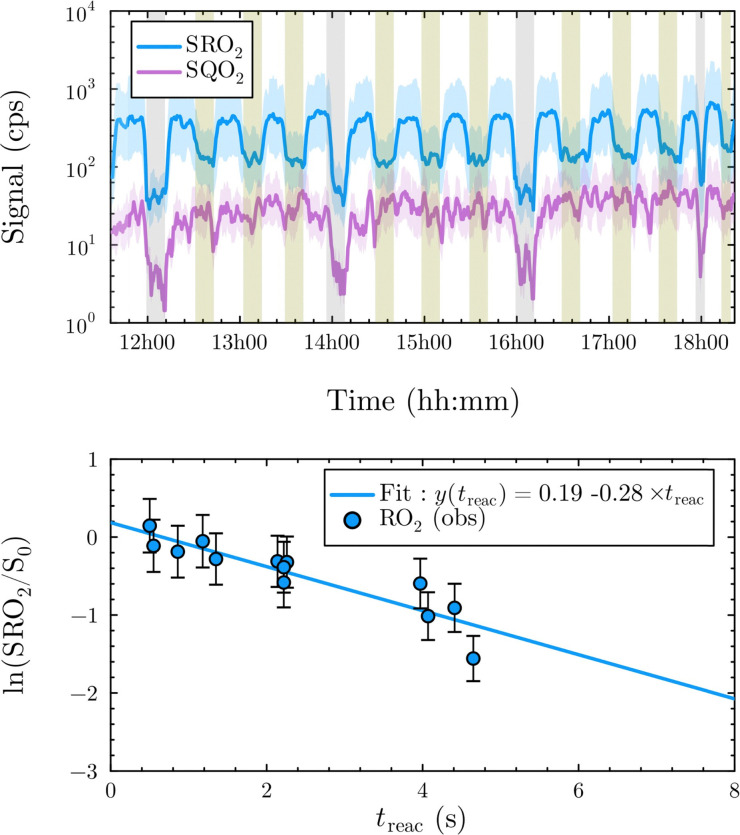
Experimental profile and kinetic analysis for 1-hexenyl-O_2_. Top: Experimental profiles for the RO_2_ (in blue) and c-QO_2_ (in purple) at *t*_reac_ = 0.5 s to 5.0 s. Green areas indicate the addition of NO in the sampling line. Gray areas indicate the photolysis lights are OFF. Bottom: Kinetic analysis: blue dots: experimental data; blue line: linear regression.

1-butenyl-O_2_, 1-pentenyl-O_2_, 1-hexenyl-O_2_, and 2-methyl-2-pentenyl-O_2_ radicals were produced photolytically in a flow reactor from their iodinated or brominated precursors, then reacted for up to 10 s in the dark before being analyzed (see Experimental section and Fig. S1, ESI†). These radicals, as well as the HOOQO_2_ and c-QO_2_ produced by the reactions and other RO_2_ potentially produced by side-reactions, were all monitored by proton transfer mass spectrometry using a chemical ionization mass spectrometer (CIMS).^20^ A list of the expected and observed ion masses (*m*/*z*) for these radicals is given in Table 1. To account exclusively for the radical signals and eliminate all potential contributions from stable compounds, excess NO (10 to 20 ppm) was added periodically at the output of the reactor, in the sampling line to the CIMS. The individual RO_2_ signals were thus determined by the difference between those recorded with “NO off” and “NO on” (Fig. 1a and Section S3, ESI†). A list of the experiments performed is given in Table S2 (ESI†). For all the RO_2_ studied except 2-methyl-2-pentenyl-O_2_ the rate coefficient for the isomerization step, *k*_iso_ (s^−1^), was determined experimentally from the observed RO_2_ decay rate, *k*^I^ (s^−1^). The contributions for the RO_2_ self- and cross-reactions with c-QO_2_, HOOQO_2_ and HO_2_ were estimated from kinetic modeling and subtracted from the overall decay rate to obtain *k*_iso_. As detailed below these contributions did not exceed 25% of the overall decay rate. In the case of 2-methyl-2-pentenyl-O_2_, neither the RO_2_ nor the expected c-QO_2_ could be observed. However, since the c-QO_2_ was observed to decompose instantly into acetone, the isomerization rate coefficient was determined from fitting the time profile of acetone to a kinetic model (see discussions in the pertaining section). The kinetic modeling was also used to study the formation of the observed stable products and propose some reaction mechanisms.

**Table 1:** Peroxy radicals studied and observed in this work, corresponding ions masses (in **bold**, those observed), and comparison between the measured and calculated rate coefficients for the unimolecular reactions

RO_2_	Expected and **observed** ions and *m*/*z*	HOOQO_2_/c-QO_2_	Observed ions and *m*/*z*	*k* ^I^ (s^−1^) measured in this work at 305 K	*k* (s^−1^) calculated in this work
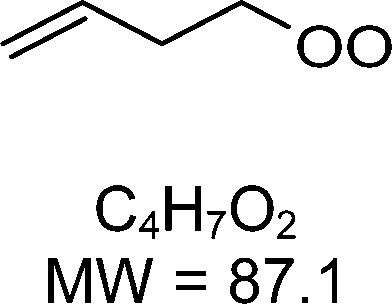	C_4_H_7_O_2_H^+^: 88.1	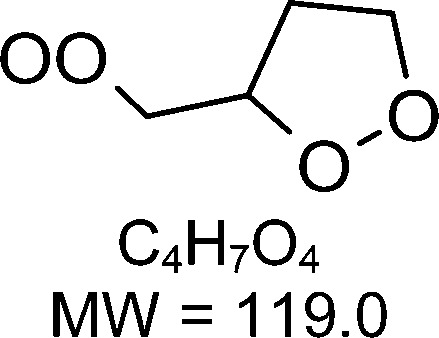	C_4_H_7_O_4_H^+^: 120.0	3.3 ± 1.5	5.3
	**C**_**4**_ **H**_**7**_ **O**_**2**_ **(H**_**2**_ **O)H** ^**+**^ **: 106.1**		**C**_**4**_ **H**_**7**_ **O**_**4**_ **(H**_**2**_ **O)H** ^**+**^ **: 138.1**		
	**C**_**4**_ **H**_**7**_ **O**_**2**_ **(H**_**2**_ **O)**_**2**_ **H** ^**+**^ **: 124.1**		**C**_**4**_ **H**_**7**_ **O**_**4**_ **(H**_**2**_ **O)**_**2**_ **H** ^**+**^ **: 156.1**		
	**C**_**4**_ **H**_**7**_ **O**_**2**_ **(H**_**2**_ **O)**_**3**_ **H** ^**+**^ **: 142.1**		**C**_**4**_ **H**_**7**_ **O**_**4**_ **(H**_**2**_ **O)**_**3**_ **H** ^**+**^ **: 174.1**		
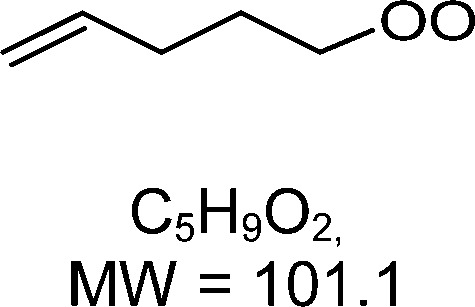	C_5_H_9_O_2_H^+^ = 102.1	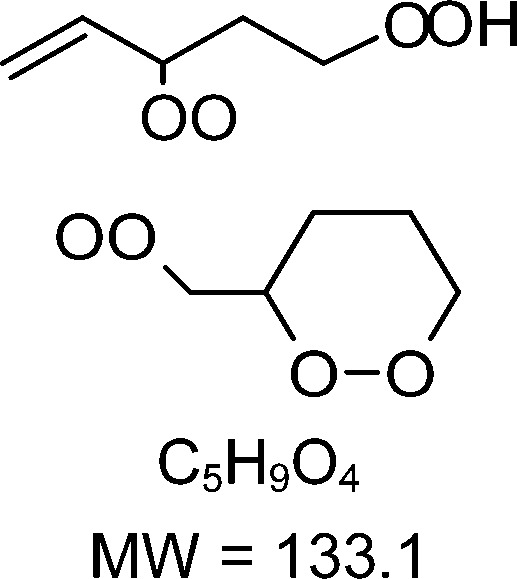	C_5_H_9_O_4_H^+^ = 134.1	0.3 ± 0.1	0.12
	**C**_**5**_ **H**_**9**_ **O**_**2**_ **(H**_**2**_ **O)H** ^**+**^ **= 120.1**		C_5_H_9_O_4_(H_2_O)H^+^ = 152.1		
	**C**_**5**_ **H**_**9**_ **O**_**2**_ **(H**_**2**_ **O)**_**2**_ **H** ^**+**^ **= 138.1**		C_5_H_9_O_4_(H_2_O)_2_H^+^ = 170.1		0.16
	**C**_**5**_ **H**_**9**_ **O**_**2**_ **(H**_**2**_ **O)**_**3**_ **H** ^**+**^ **= 156.1**		C_5_H_9_O_4_(H_2_O)_3_H^+^ = 188.1		
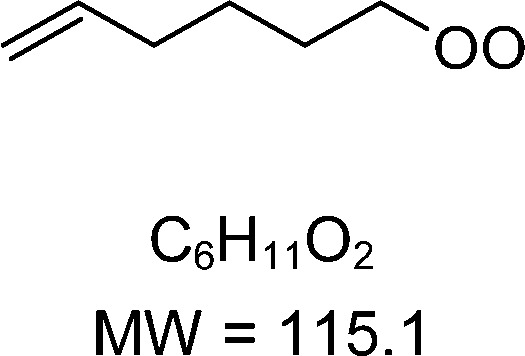	C_6_H_11_O_2_H^+^ = 116.1	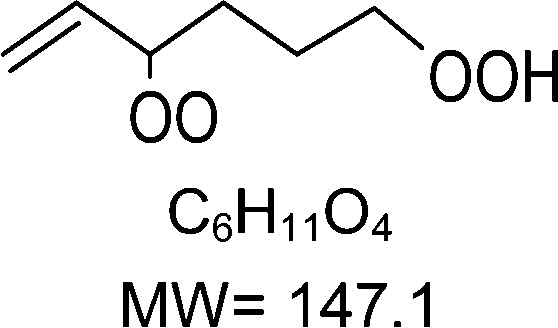	C_6_H_11_O_4_H^+^ = 148.1	0.2 ± 0.1	0.33
	**C**_**6**_ **H**_**11**_ **O**_**2**_ **(H**_**2**_ **O)H** ^**+**^ **= 134.1**		C_6_H_11_O_4_(H_2_O)H^+^ = 166.1		
	**C**_**6**_ **H**_**11**_ **O**_**2**_ **(H**_**2**_ **O)**_**2**_ **H** ^**+**^ **= 152.1**		C_6_H_11_O_4_(H_2_O)_2_H^+^ = 184.1		
	**C**_**6**_ **H**_**11**_ **O**_**2**_ **(HO**_**2**_ **)**_**3**_ **H** ^**+**^ **= 170.1**		C_6_H_11_O_4_(HO_2_)_3_H^+^ = 202.1		
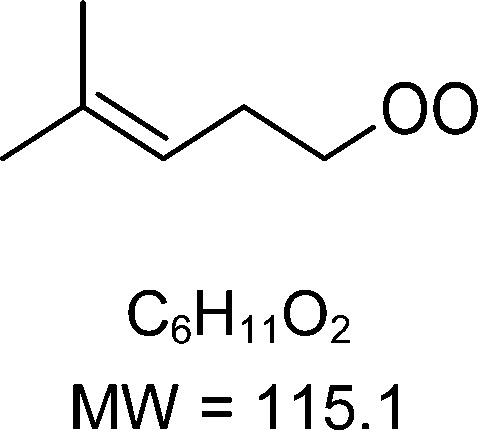	C_6_H_11_O_2_H^+^ = 116.1	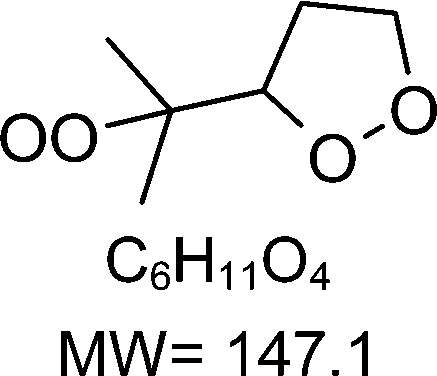	C_6_H_11_O_4_H^+^ = 148.1	≥250	266
	C_6_H_11_O_2_(H_2_O)H^+^ = 134.1		C_6_H_11_O_4_(H_2_O)H^+^ = 166.1		
	C_6_H_11_O_2_(H_2_O)_2_H^+^ = 152.1		C_6_H_11_O_4_(H_2_O)_2_H^+^ = 184.1		
	C_6_H_11_O_2_(HO_2_)_3_H^+^ = 170.1		C_6_H_11_O_4_(HO_2_)_3_H^+^ = 202.1		
CH_3_O_2_	CH_3_O_2_H^+^: 48	—	—	—	—
MW = 47	CH_3_O_2_(H_2_O)H^+^: 66				
	**CH**_**3**_ **O**_**2**_ **(H**_**2**_ **O)**_**2**_ **H** ^**+**^ **: 84**				
	CH_3_O_2_(H_2_O)_3_H^+^: 102				

### 1-Butenyl peroxy kinetics

The reaction of 1-butenyl-O_2_ was the only one for which both the RO_2_ and c-QO_2_ could be observed simultaneously (Fig. S3.1, ESI†). The overall decay for 1-butenyl-O_2_ corresponded to a first-order rate of 3.34 s^−1^. The kinetic modeling, using the rate coefficients indicated in Table S4 (ESI†), showed that the other reactions of 1-butenyl-O_2_ contributed in total to ∼2% of the overall decay. The rate coefficient reported in Table 1 is thus *k*_iso_ = 3.3 ± 1.5 s^−1^, in which the uncertainties resulted mostly from those on the small RO_2_ signals. The time profiles for the c-QO_2_ showed that this radical was indeed produced but reacted away fast (Fig. S3.1, ESI†), with an overall decay rate of ∼ 1.05 s^−1^. According to our kinetic modeling, the self- and cross-reactions of this radical with RO_2_ and HO_2_ (Table S4, ESI†) only accounted for 0.4 s^−1^ (∼ 40%) of this overall decay, thus pointing towards the existence of a unimolecular reaction with *k*^I^ = ∼ 0.9 s^−1^. This unimolecular reaction is discussed in the 1-butenyl-O_2_ mechanism section below.

### 1-Pentenyl peroxy kinetics

In the 1-pentenyl-O_2_ experiments, the RO_2_ could be observed but not the HOOQO_2_ nor the c-QO_2_ at their expected ion *m*/*z* (Fig. S3.2, ESI†). However, a significant production of CH_3_O_2_ was observed (Fig. S3.2 and Table S5d, ESI†), which is discussed in the mechanism section below. The overall decay of 1-pentenyl-O_2_ corresponded to a first-order rate of 0.39 s^−1^ (Fig. S3.2, ESI†). The kinetic model showed that its other (self- and cross-) reactions contributed together for ∼ 22% of this overall decay. Accounting for this contribution gave a value for *k*_iso_ of 0.3 ± 0.1 s^−1^ (Table 1), in which the uncertainties are mostly due to the limited reaction times investigated. The lack of observation of the HOOQO_2_ and c-QO_2_ can be in part attributed to the fast loss of the HOOQO_2_ by allyl-H-migration, with an expected rate of ∼ 48 s^−1^.^6^ For the c-QO_2_ formed from ring closure of the RO_2_ no fast loss is predicted. Its self- and cross-reactions correspond to a decay of ∼ 0.7 s^−1^, which is expected to result in a concentration that is above the detection limit for the instrument.^20,21^ (∼ 5 × 10^9^ cm^−3^, half of shown in Fig. S3.2, ESI†). Additional first-order decay of at least 1 s^−1^ would be needed to account for the absence of detection of this radical (see Fig. S3.2, ESI†).

### 1-Hexenyl peroxy kinetics

In the 1-hexenyl-O_2_ experiments a strong signal was observed for the RO_2_, but not for the expected HOOQO_2_ (or at the detection limit, Fig. 1 and S3.3, ESI†). The overall decay of 1-hexenyl-O_2_ following a first-order rate of ∼0.28 s^−1^. The kinetic simulations showed that the RO_2_ other reactions contributed to about 22% of this overall decay. Correcting the overall decay for these contributions gave a first-order rate coefficient of *k*_iso_ = 0.2 ± 0.1 s^−1^ (Table 1). The available SARs predict unimolecular loss processes for the HOOQO_2_ with rates in excess of 1 s^−1^ (see below), and its bimolecular losses are predicted by the kinetic model to be about 0.18 s^−1^. Together, these losses can potentially account for the lack of observation, though the modeling still predicts the concentration of this radical to remain somewhat above the detection limit (Fig. S3.3, ESI†).

### 2-Methyl-2-pentenyl peroxy kinetics

In the 2-methyl-2-pentenyl-O_2_ experiments neither the RO_2_ nor the expected c-QO_2_ could be observed at the expected ion *m*/*z*. With the large isomerization rate coefficient predicted by theory for this RO_2_ (∼ 266 s^−1^, Table 1), and rapid loss of c-QO_2_ producing acetone, the kinetic simulations confirmed that both the RO_2_ and c-QO_2_ concentrations should have been below the detection limit (< 1 × 10^9^ cm^−3^) within 0.25 s of entering the reaction zone, thus could not be monitored for the kinetic analysis. Since the product analysis (Product study section) indicated that the c-QO_2_ instantly decomposed into acetone, the kinetic analysis was based on the time profile for this compound, monitored at *m*/*z* 77 (*i.e.* assuming the RO_2_ isomerization step to be kinetically limiting, Fig. S3.4, ESI†). Within the uncertainties on the reaction times (± 0.1 s) the time profiles gave a lower limit for the rate of formation for acetone of 100 s^−1^, in agreement with the assumption of a near-immediate acetone formation. As discussed in the next sections, the acetone formation channel was likely to account for only ∼40% of the total decay for the c-QO_2_, therefore giving a lower limit estimate for the isomerization rate coefficient of *k*_iso_ ≥ 250 s^−1^, reported in Table 1. However, it would be interesting to study the kinetics of this fast system again with instruments that are more adapted to the relevant timescales, such as VUV photoionization or laser (for instance, fluorescence) systems.

### Theoretical determination of the rate coefficients

Vereecken *et al.* had previously provided structure–activity relationships to predict the rate of H-migration^6^ and cyclization^14^ in (un)saturated RO_2_ radicals. The cyclization rate coefficients^14^ predicted by these SAR for 1-butenyl-O_2_, 1-pentenyl-O_2_ and 2-methyl-2-pentenyl-O_2_ agreed well with the experimental observations, within a factor 1.6. However, the allyl-H-migration rate coefficients for 1-pentenyl-O_2_ and 1-hexenyl-O_2_ predicted by the SAR^6^ were almost an order of magnitude larger than those measured experimentally here. A review of the underlying source data of the SAR reveals that for allyl-H-migrations these were mostly low-level theoretical calculations, with no experimental data available for validation. New sets of theoretical calculations at a more modern level of theory were thus performed to determine the rate coefficients for all the H-migration and ring closure reactions for each parent RO_2_ radical in this work. The rate coefficients predicted for the experimental conditions studied in this work are provided in Table 1, while more details, temperature-dependent rate coefficients, and raw quantum chemical data are provided in the ESI,† and a more extensive and systematic study of the chemistry of unsaturated RO_2_, based on high-level theoretical kinetic predictions, will be reported in a companion paper updating the SAR. In addition to providing rate coefficients to compare with the experimental ones, these calculations quantified the contribution of each possible reaction channel, which can not be done experimentally with mass-spectrometry as the HOOQO_2_ formed by H-migration are isomers of the c-QO_2_ produced by ring closure reactions. The rate coefficients thus obtained theoretically agree well with the experimental measurements (see Table 1). For the 5-member cyclization of 1-butenyl-O_2_ the rate coefficient predicted by the theoretical analysis is *k*_5c_ = 5.2 s^−1^, consistent, within the uncertainties, with the rate of 3.3 (± 1.5) s^−1^ measured experimentally. For 1-pentenyl-O_2_, the theoretical study shows that this radical should undergo a 1,5 H-shift with *k*_1,5H_ = 0.12 s^−1^, and a cyclization to a 6-membered ring with *k*_6c_ = 0.16 s^−1^,^14^ thus corresponding to an overall isomerization rate coefficient of ∼ 0.3 s^−1^ (Table 1), in very good agreement with the experimental results of 0.3 (± 0.1) s^−1^. For 1-hexenyl peroxy the predicted rate coefficient for the 1,6-allylic-H-migration is *k*^I^ = 0.33 s^−1^, thus also in agreement with the experimental results of 0.2 (± 0.1) s^−1^. Finally, the rate coefficient predicted by theory for the 5-membered cyclization of 2-methyl-2-pentenyl-O_2_ of *k*_c5_ = 270 s^−1^ in good agreement with the lower limit of 250 s^−1^ obtained experimentally.

### Product study

**Fig. 2:**
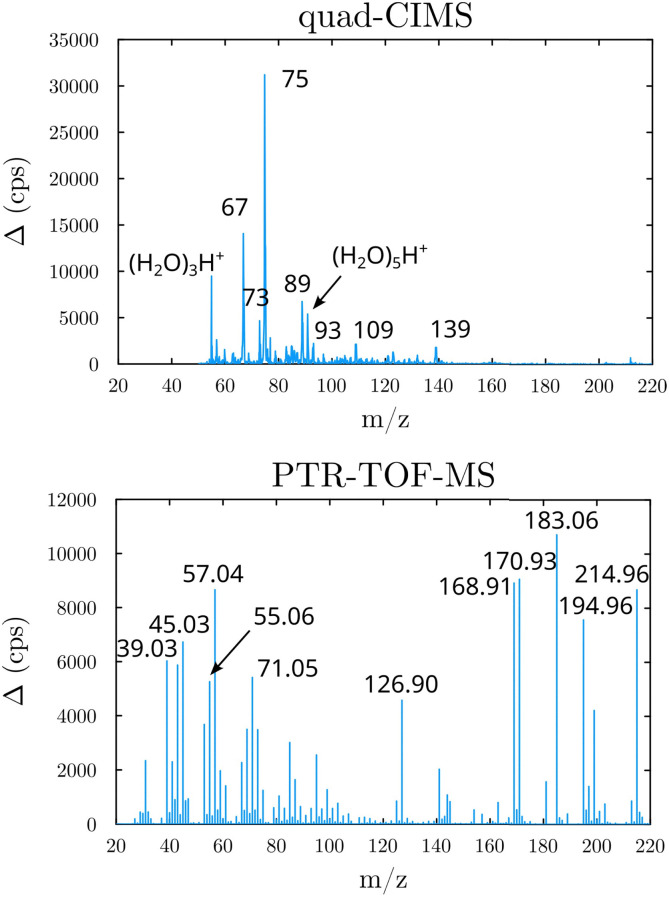
Mass spectra obtained in the 1-butenyl-O_2_ system (background subtracted, lights ON - lights OFF). Top: With the quadrupole-CIMS; Bottom: With the PTR-TOF-MS.

Beyond the prediction of the rate coefficients, the fate of the HOOQO_2_ and c-QO_2_ radicals was explored in this work to investigate the contribution of the different unimolecular pathways to the propagation or termination of the autoxidation chains. The fate of these radicals was investigated experimentally by performing product analyses both with the CIMS and with the PTR-TOFMS, both in proton transfer mode. The mass spectra and main ions observed are presented in Section S5 (ESI†), where a compound of mass M is usually detected at its water/proton clusters, *m*/*z* M + 19 and M + 37, with the CIMS and at *m*/*z* M + 1 or at ion fragments with the PTR-TOF-MS (Fig. 2).

### 1-Butenyl-O_2_ product

**Fig. 3:**
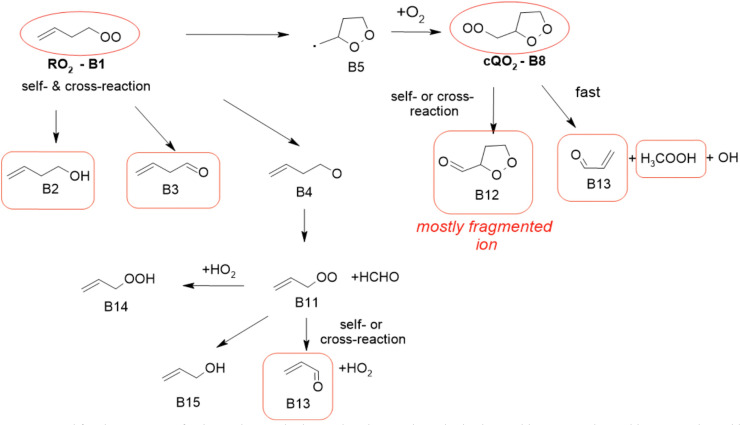
Mechanism proposed for the reactions of 1-butenyl-O_2_ radical. In red circles are the radicals observed by CIMS and in red boxes are the stable compounds observed by CIMS and PTR-TOF-MS. See the ESI† for a more extended mechanism and discussion.

As discussed in the kinetics section, both 1-butenyl-O_2_ and its expected 5- membered c-QO_2_ (B1 and B8, respectively in Fig. 3 and Table S5.2, ESI†) were observed with the CIMS. Further self- and cross reactions of the c-QO_2_ was expected to produce a cyclic alcohol (*m*/*z* 105) and a carbonyl product (*m*/*z* 103/121/139), where these reactions are predicted to contribute to the c-QO_2_ loss for about 40%. However, little signal was observed at the ion corresponding to these products (Fig. 2). However, the ion C_4_H_5_O^+^ (*m*/*z* 69, Table S5b, ESI†), was attributed to the fragmentation of the carbonyl product ion, thus suggesting that this compound was indeed produced. Using an authentic standard of *t*-butyl-OO-*t*-butyl, linear organic peroxides were found not to lead to any ion fragmentation in the CIMS, and to fragment at the O–O bond in the PTRTOF-MS, which did not correspond to the fragmentation proposed here. However, in the absence of an authentic standard for cyclic organic peroxides, it was difficult to ascertain this fragmentation and the one proposed in this work is based on the observation of similar ions or fragments in the cyclization of the other RO_2_ (see below). Under the conditions of the experiments, the self- and cross-reactions of the RO_2_ had a small contribution (∼2%) to the observed products and led to the linear carbonyl compound and alcohol observed at *m*/*z* 71/89/107 and *m*/*z* 73/91/109, respectively. The most intense ion signals observed in the experiments were *m*/*z* 57/75 (Table S5b, ESI†), which was attributed to acrolein (C_3_H_4_O), and *m*/*z* 67/85 with the CIMS and *m/z* 31 with the PTR-TOF-MS, which was attributed to methylhydroperoxide (CH_3_OOH). The identification of the latter ions as an organic hydroperoxide was based on previous calibrations of a standard of *t*-butylhydroperoxide with these instruments, showing that organic hydroperoxides were detected at *m/z* M + 18, and M + 37 with the CIMS but at the corresponding RO+ ion fragment with the PTR-TOF-MS. The large intensities observed for acrolein and methylhydroperoxide in the experiments suggested the existence of a major channel producing these compounds, complementing the c-QO_2_ self- and cross-reactions that are predicted to be the remaining c-QO_2_ loss.

### 1-Pentenyl-O_2_ products

**Fig. 4:**
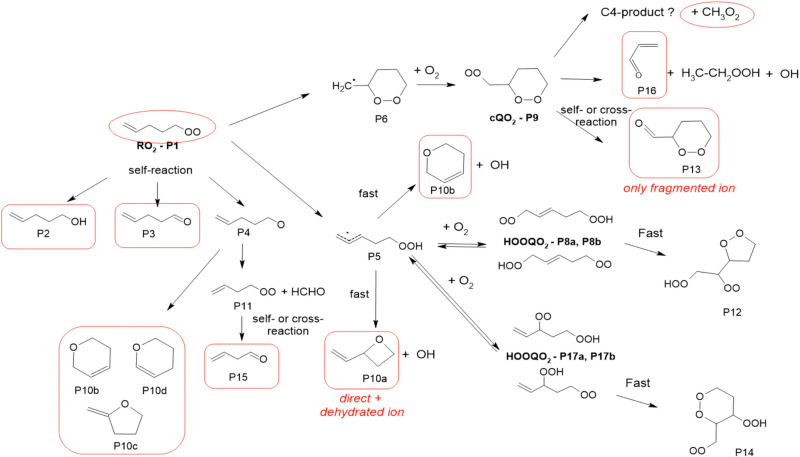
Proposed mechanism for the 1-pentenyl-O_2_ radical. See the ESI† for a more extended mechanism and discussion.

In the 1-pentyl-O_2_ experiments, the RO_2_ (P1, Fig. 4 and Table S5.4, ESI†) was observed. But neither its 6-member c-QO_2_ radical (P9) nor any HOOQO_2_ radicals resulting from its allylic 1,5 H-shift (P8/P17 in Fig. 4), both expected to be produced with comparable rates, were observed. Concerning the cyclization pathway, the heterocyclic carbonyl product (P13) expected to be produced by the self- and cross-reactions of P9 was not observed either at the expected ion (*m/z* 117/135). However, intense ion signals at *m/z* 83/101/119 were tentatively attributed to the fragmentation of the ion for this product P13, as in the 1-butenyl-O_2_ system. In the H-shift pathway, linear unsaturated carbonyl products corresponding to the HOOQO_2_ radicals P8/P17 were not observed either, but this is consistent with the existence of fast loss by ring closure channel for these radicals (see Mechanism development section and in Section S6, ESI†).

Significant signals were also observed at *m/z* 57/75/93, which were attributed to acrolein, and at *m/z* 63/81/99 with CIMS and *m*/*z* 45 with PTR-TOF-MS, which were attributed to ethylhydroperoxide. These observations suggested the existence of an unexpected yet significant reaction channel producing these two compounds. Finally, significant concentration of CH_3_O_2_ (∼1 ppb or 2.5 × 10^10^ cm^−3^, thus almost as much as the initial RO_2_) were produced in the reactions system (Fig. S4.2 and S6.1, ESI†). This formation of CH_3_O_2_ could not be accounted for with the expected mechanisms (see 1-pentenyl-O_2_ mechanism and in Section S6, ESI†) and suggests the existence of an unexpected reaction pathway. The most intense ions observed in the experiments were *m*/*z* 85/103/121, which were attributed to a cyclic ether with sum formula C_5_H_8_O (all isomers of product P10 in Fig. 4 and Table S6.4, ESI†). The formation of such a compound was supported by the observation of other intense signals at *m*/*z* 67, corresponding to the reduced ion C_5_H_6_–H^+^ and likely to result from the dehydration of the ion from the cyclic ether P10. In the absence of a standard for cyclic ethers, it was not possible to verify their ionization scheme and potential ion dehydration. However, this dehydration was supported with identical observations in the 1-hexenyl-O_2_ system (see 1-hexenyl-O_2_ mechanism). As explained in the Mechanism development section and elaborated in the ESI,† this cyclic ester was attributed to a reaction of the QOOH. Other significant ions in these experiments were *m*/*z* 87/105/123 (Table S5d, ESI†), which corresponded to the linear C5 alcohol resulting from the cross- and self-reactions of the RO_2_, although the large intensity of this ion suggests the existence of other isomers contributing to it. The corresponding carbonyl compound is an isomer of the cyclic ether proposed above, thus contributed to the large signals at *m*/*z* 85/103/121. Non-negligible signals were also observed at *m*/*z* 71, especially with the CIMS, which corresponds to the mass of the C4-carbonyl product P15 resulting from the rearrangement of the alkoxy radical P4. Alternatively, this product can also potentially be attributed to a co-product of CH_3_O_2_ in a reaction channel that remains to be identified (see 1-pentenyl-O_2_ mechanism and in Section S6, ESI†).

### 1-Hexenyl-O_2_ products

**Fig. 5:**
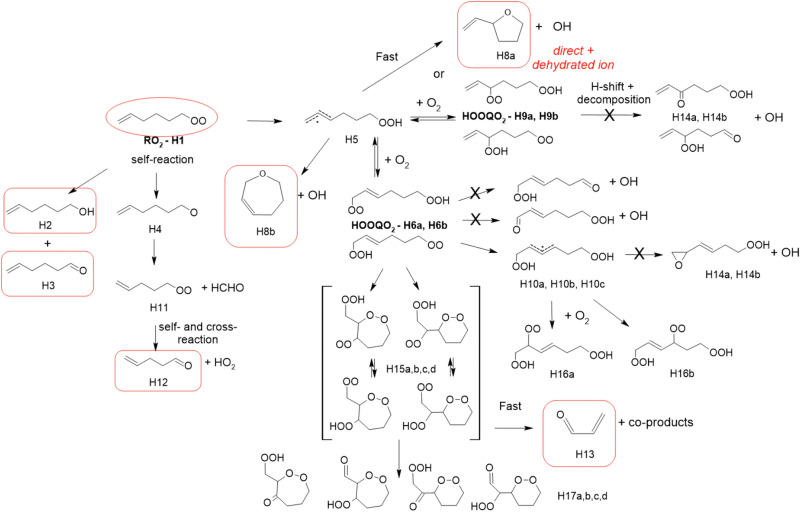
Proposed mechanism for the 1-hexenyl-O_2_ radical. See the ESI† for a more extended mechanism and discussion.

While 1-hexenyl-O_2_ (H1 in Fig. 5 and Table S5.6, ESI†) was observed in the experiments, none of the HOOQO_2_ isomers expected to result from allylic 1,6 H-shift (H6/H9 in Fig. 5) were observed. The carbonyl products of these HOOQO_2_ (H14) were not observed either. Only very little signal was observed at the ion *m*/*z* corresponding to the (HOO)_2_QO_2_ formed after one more autoxidation cycle (H-shift + O_2_ addition). Instead, intense signals were observed at *m*/*z* 99/117 and *m*/*z* 81 (Table S5f, ESI†), which were attributed (see below) to the formation of a cyclic ether of sum formula C_6_H_5_O and to the further dehydration of its ion into *m*/*z* 81, respectively. Non-negligible signals were observed at *m*/*z* 57/75 and corresponding to acrolein, and at *m*/*z* 69 and 79, which corresponded to reduced ions (Table S5f, ESI†). This indicated that, while 1-hexenyl-O_2_ itself does not undergo cyclization, some of the resulting HOOQO_2_ isomers might (see mechanistic discussion). The resulting c-QO_2_ radicals would thus react by different channels, producing either acrolein or heterocyclic carbonyl compounds, mostly detected at their fragment ions, as observed in the 1-butenyl-O_2_ and 1-pentenyl-O_2_ systems. The signals observed at *m*/*z* 85/103, were attributed to the linear C5 carbonyl compound H12 produced by the further rearrangement of the alkoxy radical, H4, produced by the RO_2_ (H1) self-reaction.

### 2-Methyl-2-pentenyl-O_2_ products

In the 2-methyl-2-pentenyl-O_2_ system, neither the RO_2_ nor the c-QO_2_ could be observed, and only very little signal was observed at the expected ion for the heterocyclic carbonyl compound produced by the self- and cross-reaction of the c-QO_2_ (MP8, *m*/*z* 117/135). By analogy with the observations in the 1-butenyl-O_2_ and 1-pentenyl-O_2_ systems, the ion for MP8 was expected to fragment into C_5_H_7_O^+^, *m/z* 83. This ion overlapped with the most intense signals observed in the experiments, largely due to the brominated precursor (*m*/*z* 162), producing intense ions at *m*/*z* 83, 163 and 165 even in the dark. However, the differential spectra (“lights ON” – “lights OFF”) in Fig. S5g (ESI†) suggest that this ion, and thus the corresponding product MP8, was produced in large concentrations in the reaction: the signal strength of 82 000 Hz in the CIMS measurement corresponds to at least 8 ppb (or 2 × 10^11^ cm^−3^) assuming a detection sensitivity of up to 10 000 Hz ppb^−1^. In addition, large concentrations of CH_3_O_2_ radicals (MP9), ∼2 ppb (or 5 × 10^10^ cm^−3^), thus of the same order than the initial c-QO_2_ concentration, were observed with the CIMS at *m*/*z* 66/84, and is a likely co-product of this heterocyclic carbonyl product. The next most intense ion signal observed in these experiments was *m/z* 59, which was attributed to acetone and indicated the existence of a major production pathway for this compound in this system. Some significant signals were also observed for acrolein, which was a likely co-product of acetone in a reaction channel similar to those observed with the c-QO_2_ from 1-butenyl-O_2_ and 1-pentenyl-O_2_. Smaller signals at *m/z* 99, were tentatively attributed to the linear carbonyl product from the self- and cross-reaction of the RO_2_.

### Mechanism development

**Fig. 6:**
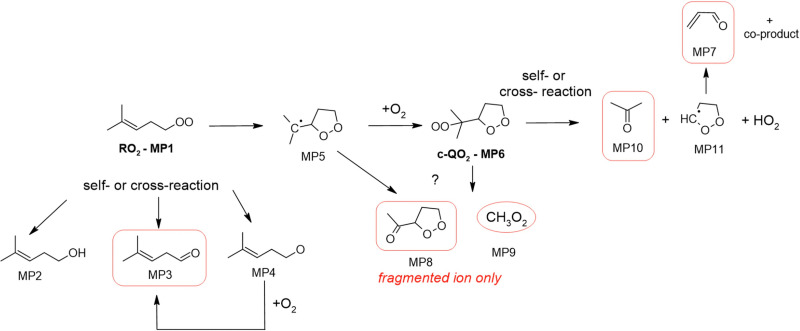
Proposed mechanism for the 2-methyl-2-pentenyl-O_2_ radical. See the ESI† for a more extended mechanism and discussion.

Mechanisms describing the fate of the radicals studied are proposed in this work based on the observed ions, available experimental and theoretical data on RO_2_ reactivity, and structure–activity relationships for the rate coefficients of the relevant reactions. Simplified mechanisms for each of the radicals studied are shown in Fig. 3–6, in which the observed compounds are circled in red. These figures and the discussion below are accompanied by additional material in Section S6 of the ESI,† including additional theoretical calculations of specific reactions of importance in the degradation mechanism, with particular emphasis on the fragmentation of cycloperoxides, and the fate of the intermediates after allylic H-migration (formation of cyclic ethers). Section S6 (ESI†) also presents extended reaction mechanisms for each of the intermediates with detailed discussions in Schemes S1 to S12 (ESI†). In addition to the qualitative mechanisms proposed in Fig. 3–6 and Schemes S1–S12 (ESI†), some kinetic simulations of the main product pathways were performed to compare semi-quantitatively with the observed products. Details on these simulations are given in Section S4 (ESI†), including the full list of reactions and rate coefficients used, including the photolytic reactions taking place only in the irradiation zone of the reactor and bimolecular and unimolecular reactions for the RO_2_ (including the HOOQO_2_ and c-QO_2_), taking place both in the irradiation zone and in the dark reaction zone of the reactor. As indicated in Section S4 (ESI†), the rate coefficients for the bimolecular reactions were taken from the IUPAC database^26^ and Jenkin *et al.*^27^ Rate coefficients for allylic H-migration in the primary RO_2_ were calculated theoretically in this work, while rates for cyclization and for H-migration in secondary RO_2_ are taken from the SARs by Vereecken *et al.*^6,14^ The isomerization and dissociation of alkoxy radicals, RO, are modeled using the SARs by Vereecken and Peeters,^28^ and Novelli *et al.*^29^ The O_2_ addition on alkyl radicals was assumed to be fast enough (rates ≥ 10^7^ s^−1^ under atmospheric conditions) to preclude any competing reactions. The O_2_ addition on allylic radicals, however, is reversible, allowing the allyl–O_2_ peroxy radicals to isomerize.^15,30^ In the current work the reaction rate coefficients for the allylic RO_2_ were assumed to be of the same order of magnitude as for isoprene-RO_2_, with ∼10^6^ s^−1^ for O_2_ addition (assuming [O_2_] = 5 × 10^18^ cm^−3^ in air) and redissociation rates between 0.01 s^−1^ and 25 s^−1^. Thus, in the sections below only the main points are discussed. The reader is referred to Section S6 of the ESI† for more detail.

### Fate of c-QO_2_ radicals

A consistent feature observed with the unsaturated RO_2_ reacting through ring closure and forming c-QO_2_ (1-butenyl-O_2_, 1-pentenyl-O_2_) is the formation of acrolein. In the ESI† we examine several potential formation pathways for this product, but no plausible one could be found. Instead, the currently known chemistry produces alkoxy radicals in c-QO_2_ self- and cross-reactions, which decompose to a carbonyl product (*e.g.* acetone for 2-Me-2-pentenyl-O_2_) with a C3 coproduct O=CHCH_2_CH=O (malondialdehyde in keto/enol equilibrium with hydroxy-acrolein, O=CHCH=CHOH). We refer to the ESI† for a detailed discussion of the potential formation pathways explored.

### Fate of allylic HOOQ/HOOQO_2_ intermediates

A consistent feature observed for unsaturated RO_2_ reacting by allylic H-migration and forming HOOQO_2_ is the presence of intense signals corresponding to mono-oxygenated products, *i.e.* an unsaturated carbonyl or cyclic ether with the same number of carbons and hydrogens as the primary RO_2_. In the ESI,† numerous potential formation mechanisms for such products are examined, most of them involving an alkoxy radical intermediate, and some requiring (significant) chemical activation of the intermediates. For a mechanism based on cyclization of unsaturated alkoxy radicals, tentative supporting observations were found (see below and Section S6, ESI†), but the available data does not allow unequivocal determination of the mechanism at play; some of the observations suggest distinct isomers are formed (see Section S6, ESI†). In the mechanisms below, we therefore can not explain these products in a satisfactory manner and refer to the ESI† for a more extensive discussion.

### 1-Butenyl-O_2_ mechanism

A mechanism describing the reactions of 1-butenyl-O_2_ is presented in Fig. 3. In this mechanism, the main channel is the 5-membered cyclization of 1-butenyl-O_2_ (B1) producing a cyclic alkyl radical (B5), then, after addition of O_2_, a c-QO_2_ (B8). The product study is consistent with the self-reaction of the c-QO_2_ producing a cyclic carbonyl product (B12). However, the observation of acrolein (B13) as a major reaction product in the experiments suggested the existence of an important reaction channel producing this compound. Under the conditions of the experiments, the HO_2_ and self- and cross-reaction of the c-QO_2_ corresponded to a first-order rate of ∼0.4 s^−1^, while its observed decay rate was of the order of 1.1 s^−1^ (Fig. S3.1 (ESI†) and kinetic discussion 1-butenyl peroxy kinetics above). These reactions, however, do not form acrolein. Its formation was thus attributed to an unknown channel. Although the exact mechanism for this channel remains unclear, it is supported by similar observations with the c-QO_2_ from 1-pentenyl-O_2_ (see 1-pentenyl-O_2_ mechanism). As indicated by the reactions rates above, under the conditions of our experiments the channel producing the cyclic carbonyl (B12) represented only 31% of the fate of the c-QO_2_, while the channel producing acrolein represented 69%. The mechanism also shows a channel producing acrolein from the rearrangement of the alkoxy radicals (B4) from the initial RO_2_, but its expected yield is minor as the contribution of RO_2_ + RO_2_ to the overall reaction of 1-butenyl-O_2_ is small.

### 1-Pentenyl-O_2_ mechanism

The mechanism proposed for 1-pentenyl-O_2_ is presented in Fig. 4. Besides the self- and cross-reactions (P1), the main two unimolecular reaction pathways for 1-pentenyl-O_2_ are the cyclization into the alkyl radical (P6) leading, after the addition of O_2_, to the c-QO_2_ (P9) and the 1,6 H-shift into the HOOQ radical (P5), both pathways having comparable rates. For the cyclization pathway, while the c-QO_2_ (P9) was not observed, the formation of the heterocyclic carbonyl product (P13) by its self-reaction was supported by the observation of its fragment ion. In addition, the significant presence of acrolein and ethylhydroperoxide suggested the existence of another significant reaction channel producing these compounds. As discussed in the kinetic section, the lack of observation of the c-QO_2_ suggested a decay rate for this radical of at least 1 s^−1^, while its self- and cross-reactions corresponded to a first-order rate of only ∼ 0.17 s^−1^ under the experimental conditions (assuming the RO_2_ and c-QO_2_ concentrations given in Fig. S3.2 and rate coefficients in Table S4, ESI†). This suggested that the unexpected reaction channel was unimolecular rather than bimolecular. Although the exact mechanism of this channel is still unclear, we propose, as with 1-butenyl-O_2_, a direct decomposition of the c-QO_2_ into acrolein and ethylhydroperoxide. Finally, the substantial formation of CH_3_O_2_ indicated the potential occurrence of a third reaction channel for the c-QO_2_, which is proposed to produce CH_3_O_2_ and a C4 co-product. The allylic H-shift pathway in the primary RO_2_ was expected to produce the alkyl radical (P5), which after allylic rearrangement and O_2_ addition, should have produced two sets of HOOQO_2_ radicals, with the double bond in C1 and C2, respectively: (P8a)/(P8b) and (P17a)/(P17b) (Fig. 4). Within each set of HOOQO_2_, rapid scrambling reactions (1 to 100 s^−1^) would result in two different isomers. Stable carbonyl products formed from these HOOQO_2_ radicals were not observed. Instead, intense signals indicated the existence of an unexpected but important channel producing a cyclic ether (P10a/P10b or other isomers). Both the sum formula for this cycloether and the intensity of the signals suggested that it was produced directly from the QOOH rather than from the HOOQO_2_ radicals or by bimolecular reactions. Even though such unimolecular reaction of the QOOH would be in strong competition with O_2_ addition, the reversibility of the latter would allow access to a fast subsequent cyclization. Such a unimolecular cyclization of a QOOH was further supported by identical observations in the 1-hexenyl-O_2_ system (see 1-hexenyl-O_2_ mechanism). The ESI† has a more extensive discussion on the formation pathways to cyclic ethers.

### 1-Hexenyl-O_2_ mechanism

A mechanism for 1-hexenyl-O_2_ is presented in Fig. 5. This radical is expected to undergo almost exclusively a 1,6 H-shift to produce the HOOQ radical (H5). Allylic rearrangement and O_2_ addition should, then, produce two sets of HOOQO_2_, *i.e.* (H9a)/(H9b), with the double bond on carbon C1, and (H6a)/(H6b) with the double bond on carbon C2, represented in the mechanism. Fast scrambling reactions exchanging an H-atom between the hydroperoxyl and peroxy sites should result in two isomers for each HOOQO_2_. None of these HOOQO_2_ nor their carbonyl products were observed in the experiments. As discussed in detail in the ESI,† the P5-derived HOOQO_2_ are expected to be lost by a fast ring-closure reaction, forming a HOO-cQO_2_ (shown as the various H15 isomers in Fig. 5). Additionally, intense signals indicated the formation of large amounts of the cyclic ethers C_6_H_5_O (H8a/H8b and isomers), mostly observed at their dehydrated ion (*m/z* 81, the most intense signals observed in these experiments). As in the 1-pentenyl-O_2_ system, both the sum formula for this cycloether and the large amount produced suggested a direct, unimolecular production from the HOOQ (H5) and/or HOOQO_2_ (P8/P17). The observation of acrolein and fragment ions at *m/z* 69 and 79 in the product analysis suggested also that a fraction of the HOOcQO_2_ (P17) formed from P8/P17 did undergo fragmentation producing acrolein, as already discussed for the 1-butenyl-O_2_ and 1-pentenyl-O_2_ systems.

### 2-Methyl-2-pentenyl-O_2_ mechanism

The proposed mechanism for 2-methyl-2-pentenyl-O_2_ is presented in Fig. 6. As shown in this mechanism, its main expected reaction pathway is the cyclization into the alkyl radical (MP5) and potential formation of the c-QO_2_ (MP6). Further self- and cross reactions of the c-QO_2_ should produce an alkoxy radical, which should readily decompose into acetone and the cyclic radical (MP11). The latter is expected to undergo ring opening (perhaps related to what was observed with the c-QO_2_ from 1-butenyl-O_2_ and 1-pentenyl-O_2_) to form O=CHCH_2_CH=O (see above, and ESI†). The observation of very intense ion signals potentially corresponding to the dehydrated ion of the heterocyclic carbonyl product (MP8) suggest that another reaction channels produces this compound, although this is not expected from the decomposition of the alkoxy radical from (MP6). Finally, large concentrations of CH_3_O_2_ radicals (MP9) were also observed to be produced in the experiments, thus confirming the existence of another, yet unidentified reaction channel. The relative signals of acetone (12 000 Hz) and CH_3_O_2_ (11 000 Hz) combined with their relative detection sensitivities (*i.e.* ∼ 5000 Hz ppb^−1^ for CH_3_O_2_^20^ and 7000 Hz ppb^−1^ to 10 000 Hz ppb^−1^ for acetone) suggested that the acetone channel contributed for 35 to 44% and the CH_3_O_2_ channel for 56 to 65% of the c-QO_2_ decomposition. For this reason, a correction factor of 0.4 is applied in the determination of the isomerization rate coefficient for this radical in the kinetic analysis.

## Conclusion

This study investigated the rate coefficients and products of the unimolecular reactions of a series of unsaturated RO_2_. The rate coefficients, determined in most cases from the direct observation of the RO_2_ decay, match very well the theoretical kinetic predictions at a high level of theory, providing mutual support. Although the experiments could not fully speciate the products, the excellent agreement with theory suggests that the observed reactions indeed correspond to the theoretically proposed mechanisms, *i.e.* ring closure *vs.* allylic H-migrations. In a second part, the ions and products observed experimentally were compared to detailed oxidation mechanisms derived from explicit theoretical calculations, structure–activity relationships, and literature data. Many of the proposed reaction products and channels can not be explained by known mechanisms, thus showing that our understanding of these mechanisms is still highly incomplete. In particular, the carbonyl products expected from the c-QO_2_ radicals could not be observed, or mostly as dehydrated ion, while intense signals were observed which were attributed to acrolein in the 1-butenyl-O_2_ and 1-propenyl-O_2_ systems, and to acetone in the 2-Me-2-pentenyl-O_2_. These products suggest that the cycloperoxide-alkylperoxy radicals formed after cyclization of the unsaturated RO_2_ rapidly decompose. However, the observed rate of product HOOQO_2_ or c-QO_2_ decomposition is not always in agreement with the available literature data on unimolecular or bimolecular reactions of the HOOQO_2_ intermediates, nor is there a clear formation path for acrolein in these systems. Secondly, for the unsaturated RO_2_ subject to allylic H-migration, *i.e.* 1-pentenyl-O_2_ and 1-hexenyl-O_2_, the largest signals observed in the experiments corresponded to cyclic ethers (different isomers + dehydrated ions). The formation of these compounds is not compatible with the currently available literature. Several pathways for both these subsequent chemistries are discussed, but at this time we are unable to elucidate how this chemistry takes place. The observation of prominent contributions of termination reactions to the autoxidation chain after both cyclization and allylic-H-migration in unsaturated RO_2_ raises the question whether double bonds are favorable for autoxidation to highly oxidized molecules, and whether these functionalities might not instead be a hindrance to autoxidation. This goes against the current consensus in the literature, in which double bonds are thought to be the promotors of autoxidation, and cyclization and allylic-H-migration as the necessary steps to oxidize all the carbon atoms and reach the observed O:C ratios.

## Author contributions

BN: conceptualization (experimental), methodology (experimental), investigation (experimental), formal analysis, funding acquisition, project administration, supervision, writing – original draft. LV: conceptualization (theoretical), methodology (theoretical), investigation (theoretical), formal analysis, writing – original draft.

## Data availability

The experimental data (MS Excel spreadsheet) will be made available and sent to the readers upon request. The quantum chemical data for these calculations are available as a textfile in the repository at URL https://doi.org/10.26165/JUELICH-DATA/ZGIZV3. This repository contains geometries, rotational constant, vibrational wavenumbers, and energies at various levels of theory. 

## Conflicts of interest

There are no conflicts to declare.

## Supplementary Material

CP-026-D4CP02718C-s001
