# Supplementary material for: H-shift and cyclization reactions in unsaturated alkylperoxy radicals near room temperature: propagating or terminating autoxidation?
Source: Phys Chem Chem Phys. 2024 Sep 16;26(39):25373–84. doi: 10.1039/d4cp02718c (PMC11418804; doi:10.1039/d4cp02718c)
Supplement: CP-026-D4CP02718C-s001 [file CP-026-D4CP02718C-s001.pdf]

Supplementary Information (SI) for Physical Chemistry Chemical Physics.
This journal is © the Owner Societies 2024

Supporting Information
©Wiley-VCH 2021
69451 Weinheim, Germany

H-shift and Cyclisation Reactions in Unsaturated Alkylperoxy Radicals near Room Temperature: propagating or terminating autoxidation ?

Barbara Nozière,* and Luc Vereecken*

Table of Contents

Figure S1. Schematics of the experimental set-up 3
Table S2. List of the experiments 4
Section S3. Kinetic profiles and analysis 5
Section S4. Kinetic modeling 9
Table S4. List of the reactions and rate coefficients used in the kinetic modeling 9
Section S5. Mass spectra and main ions detected in the experiments 11
Section S6. Mechanistic considerations 19
Decomposition of c-QO_2 and formation of acrolein 19
Formation of unsaturated cyclo-ethers, carbonyls, alcohols, or epoxides 20
Mechanism A: carbonyl channel in primary RO_2+RO_2 21
Mechanism B: cycloether+OH formation from HOOQ 21
Mechanism C: cyclisation of primary alkoxy radicals, followed by HO_2 elimination 22
Mechanism D: cyclisation of hydroperoxyalkoxy radical HOOQO, followed by HO_2 elimination 24
Mechanism E: unknown subsequent chemistry of $(HOO)_2QO_2$ 24
Mechanism F: Chemistry induced by protonation in the ionization chamber 25
Formation of alkylhydroperoxides, CH_3OOH and C_2H_5OOH 25
1-butenyl-O_2 annotated mechanism 27
1-pentenyl-O_2 annotated mechanism 28
1-hexenyl-O_2 annotated mechanism 31
2-Me-2-pentenyl-O_2 annotated mechanism 33
Section S7. Rate coefficients from theoretical calculations 34
Table S7.1 34
Table S7.2. 35
Section S8. References 36

Figure S1. Schematics of the experimental set-up

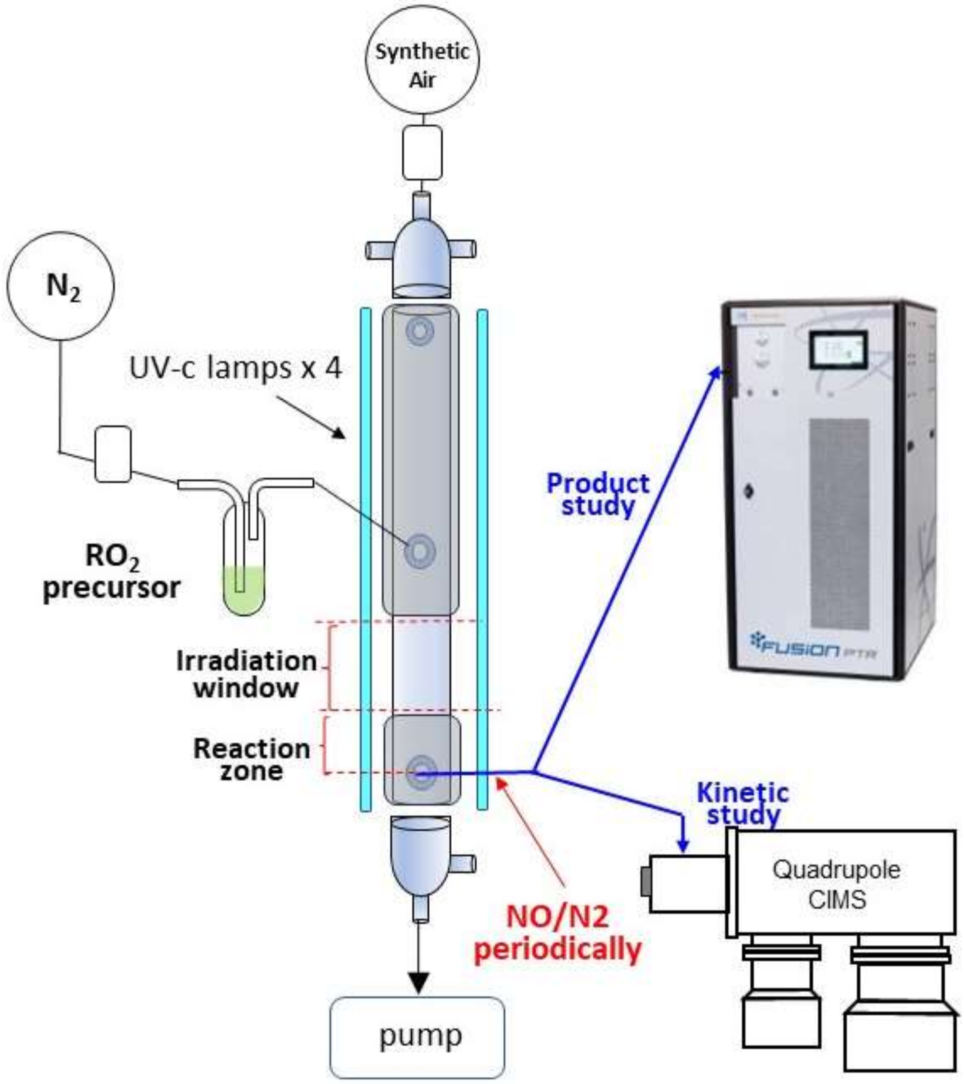

Table S2. List of the experiments

Date	[precursor] (ppm)	Fair (sLm)	t_{reac} (s)	Type of experiment
**1-butenylO$_2$**, *$[RO_2]_o$ = 4.8 ppb (1.2×10^{11} cm^{-3})				
19/05/23	1.1	3	1.4	Kinetics
22/05/23	1.1	3	1.4	Kinetics
29/05/23	1.1	3	0.7 - 1.0	Kinetics
30/05/23	1.1	3	0.3 – 2.4	Kinetics
31/05/23	1.1	3		Calibration
21/06/23	1.6	3	0.5	Kinetics
22/06/23	2.2	3	0.3 – 0.5	Kinetics
26/06/23	2.2	3	0.3	Product study
**1-pentenylO$_2$**, *$[RO_2]_o$ = 1.5 ppb (3.8×10^{10} cm^{-3})				
30/06/23	0.05	3	0.3	Kinetics
05/07/23	0.05	3	0.3	Kinetics
10/07/23	0.05	3	0.7-1.1	Kinetics
13/07/23	0.05	3	0.3 – 1.1	Kinetics
01/08/23	0.05	3		Product study
**1-hexenylO$_2$,** *$[RO_2]_o$ = 2.2 ppb (5.5×10^{10} cm^{-3})				
11/04/23	0.9	3.75	0.1	Kinetics
13/04/23	1.1	2.5	3.6	Kinetics
14/04/23	0.8 – 1.8	1.4 – 3.2	1.3 – 6.2	Kinetics
17/04/23	1.3	1.1	0.1	Kinetics
19/04/23	0.6	1.1	0.1 – 8.7	Kinetics
24/04/23	0.2	3	3.2	Kinetics
25/04/23	0.3	3	0.3 – 6.8	Kinetics
04/05/23	1.0	1	3.8 – 9.0	Kinetics
10/05/23	0.8	3	0.5 – 4.9	Kinetics
12/05/23	0.8	3	1.4 – 8.4	Kinetics
17/05/23	0.8	3	3.5	Calibration
27/06/23	0.8	3	1.5	Product study
**2 methyl 2-pentenylO$_2$,** *$[RO_2]_o$ = 0.3 ppb (7.1×10^{9} cm^{-3})				
14/07/23	8.7 -10.4	2.5 - 3	0.3 – 0.4	Kinetics
17/07/23	10.2 – 15.1	1 - 2	0.4 – 1.7	Kinetics
25/07/23	18.3	1	1.65	Product study

*Typical concentration estimated at the entrance of the reaction zone.

Section S3. Kinetic profiles and analysis

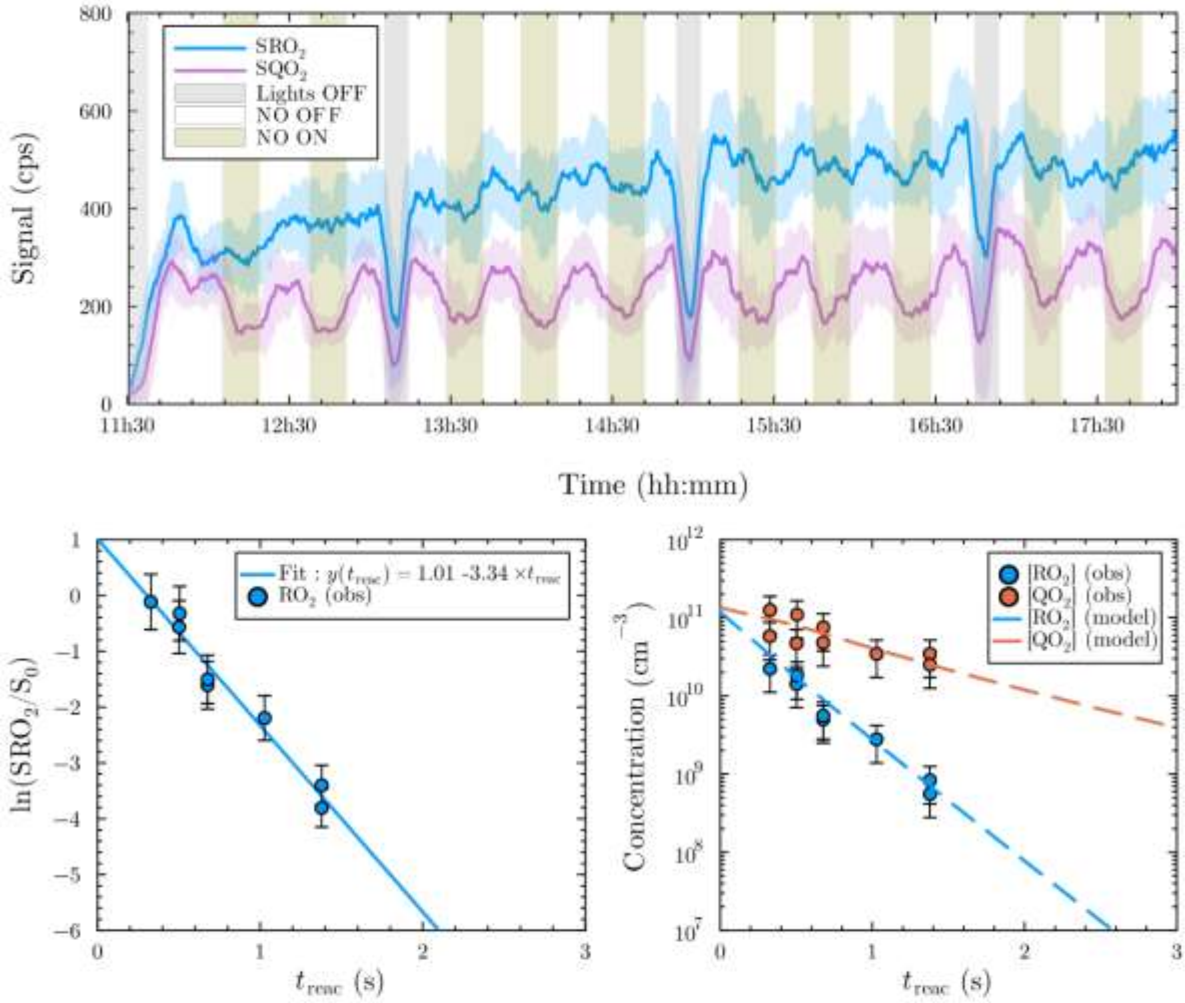

Figure S3.1: Kinetic analysis for 1-butenyl-O_2. Top: Experimental profiles, blue line = RO_2, pink line = c-QO_2; Middle: linear regression on the overall RO_2 decay; Bottom: comparison with the kinetic model.

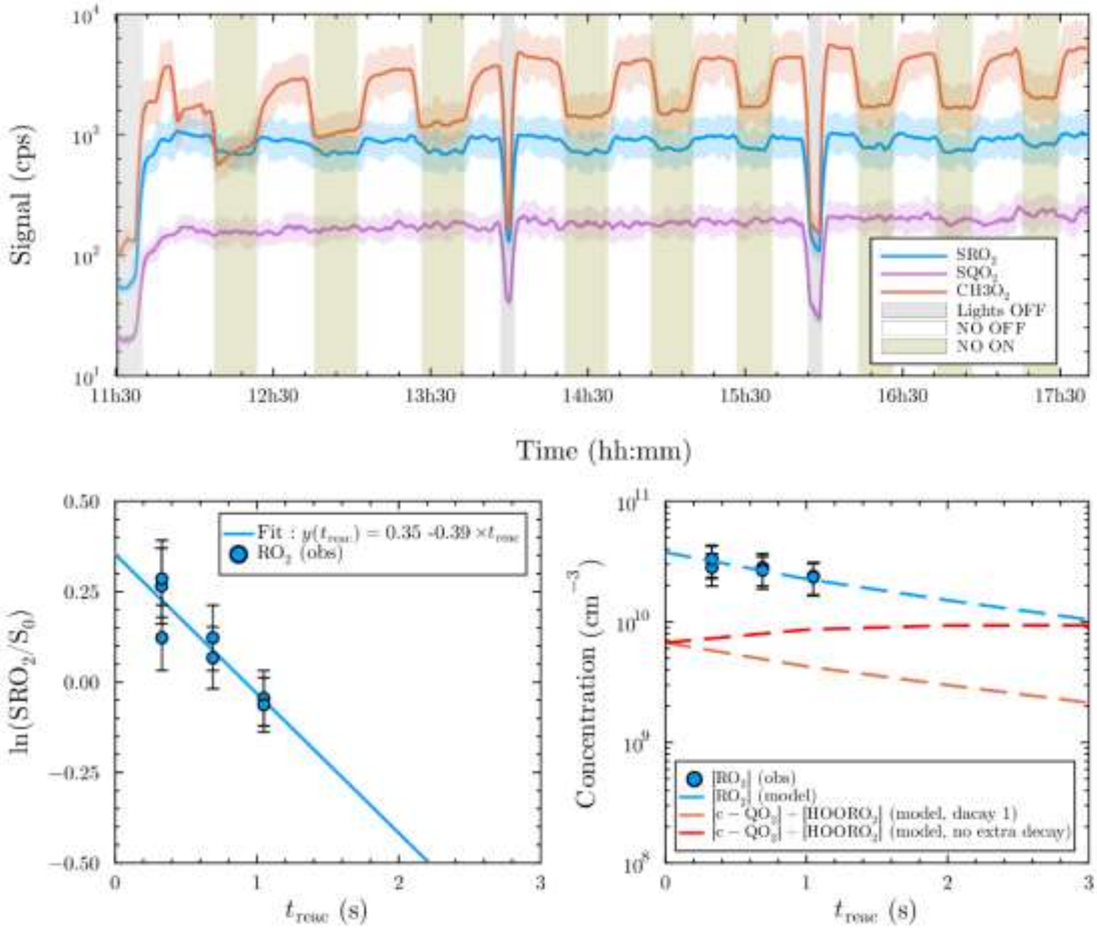

Figure S3.2: Kinetic analysis for 1-pentenyl-O_2. Top: Experimental profiles: blue line = RO_2, pink line = c-QO_2 + sum of all $HOOQO_2$, red line = CH_3O_2; Middle: linear regression on the RO_2 overall decay; Bottom: comparison with the kinetic model.

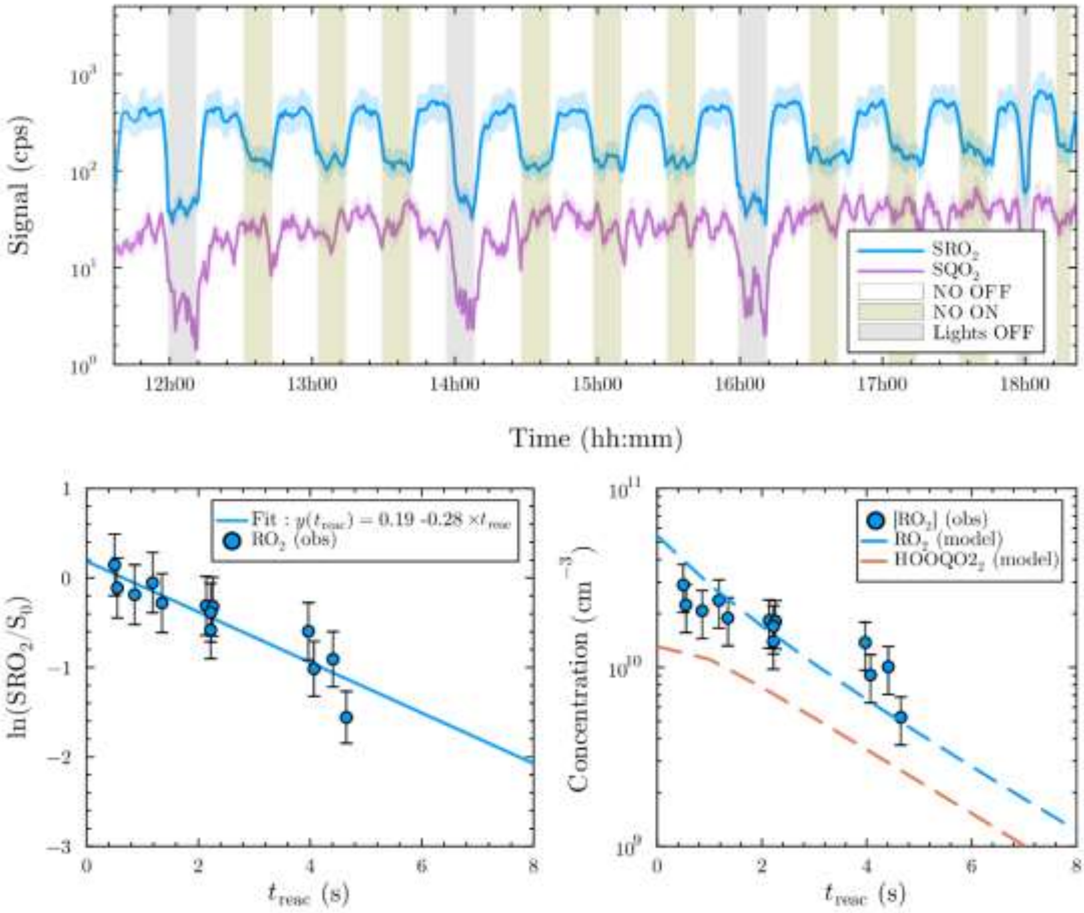

Figure S3.3: Kinetic analysis for 1-hexenyl-O_2; Top: Experimental profiles; blue line = RO_2, pink line = sum of all $HOOQO_2$: Middle: linear regression on the overall RO_2 decay; Bottom: comparison with the kinetic model.

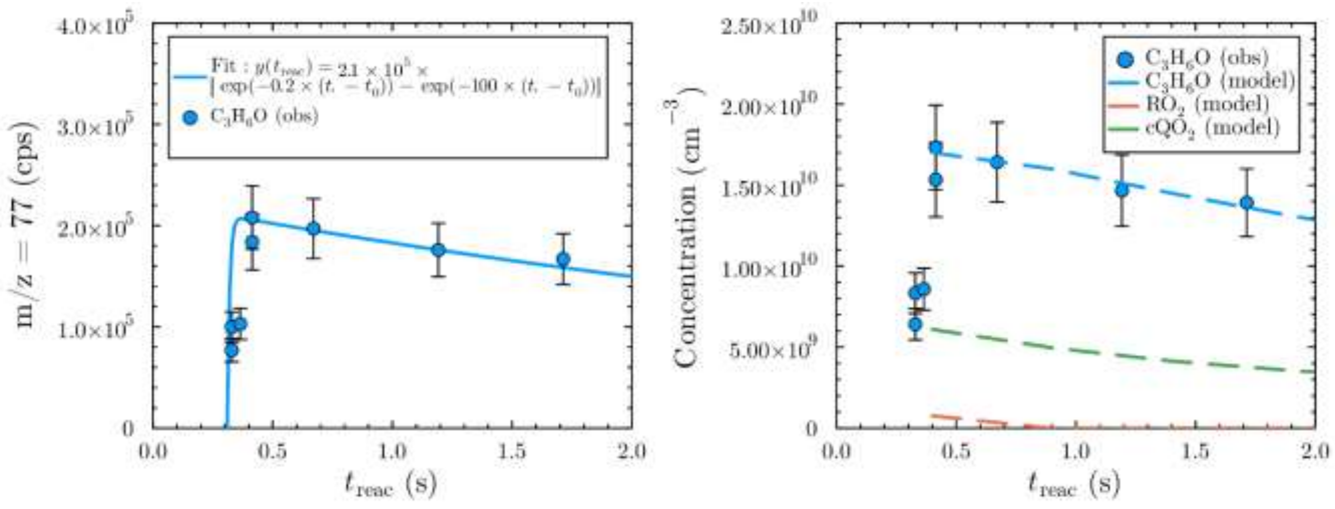

Figure S3.4: Kinetic analysis for 2 methyl-2-pentenyl peroxy.

Section S4. Kinetic modeling

Kinetic modeling was performed using the ChemSimul V3.90 software to calculate the RO_2, $HOOQO_2$ and c-QO_2 concentrations and compare them with the observed time profiles. These simulations also allowed to estimate the contributions of bimolecular and other reactions to the overall RO_2 decays in the kinetic analysis. The modeling was performed in two steps: first, the concentrations of RO_2, $HOOQO_2$ and c-QO_2 produced photolytically in the irradiation region were determined. In a second step these predicted concentrations were used as initial values to simulate the reactions taking place in the reaction region in the dark.

Table S4. List of the reactions and rate coefficients used in the kinetic modeling

Reaction	units	1-butenyl-O_2	1-pentenyl-O_2	1-hexenyl-O_2	2-methyl-2-pentenyl-O_2
(1) R-I + hν → R + I	s^{-1}	2×10^{-2} *	2×10^{-2} *	2×10^{-2} *	/
(1b) R-Br + hν → R + Br	s^{-1}	/	/	/	2×10^{-3} (a)
(2) O_2 + hν → O + O	s^{-1}	2×10^{-9} (b)	2×10^{-9} (b)	2×10^{-9} (b)	2×10^{-9} (b)
(3) RO_2 + hν → RO + O	s^{-1}	2×10^{-3} (c)	2×10^{-3} (c)	2×10^{-3} (c)	2×10^{-3} (c)
(4) $HOOQO_2$ / c-QO_2 + hν → HOOQO / c-QO + O	s^{-1}	2×10^{-3} (c)	2×10^{-3} (c)	2×10^{-3} (c)	2×10^{-3} (c)
(5) HO_2+ hν → OH + O	s^{-1}	2×10^{-3} (c)	2×10^{-3} (c)	2×10^{-3} (c)	2×10^{-3} (c)
(6) O_3 → O_2 + O	s^{-1}	0.2	0.2	0.2	0.2
(7) $H_3CC(O)CH_3$ → CH_3 + $H_3CC(O)$	s^{-1}				0.1
(8) ROOR′ +hν → RO + OR′	s^{-1}	4×10^{-4} (d)	/	/	4×10^{-4} (d)
(9) ROOH (incl $HOOQO_2$) +hν → RO+OH	s^{-1}		4×10^{-4} (e)	4×10^{-4} (e)	
(10) R + O_2 + M → RO_2 + M	$cm^3\ s^{-1}$	5×10^{-12} (f)	5×10^{-12} (f)	5×10^{-12} (f)	5×10^{-12} (f)
(11) O_2 + O + M → O_3 + M	$cm^3\ s^{-1}$	1.4×10^{-14}(g)	1.4×10^{-14} (g)	1.4×10^{-14} (g)	1.4×10^{-14}(g)
(12) RO_2 + RO_2 → 2 RO + O_2 / RH + R=O	$cm^3\ s^{-1}$	6.7×10^{-13}(h)	1.1×10^{-12} (h)	1.4×10^{-12} (h)	1.4×10^{-12} (h)
(13) $HOOQO_2$ (or c-QO_2) + $HOOQO_2$ → products	$cm^3\ s^{-1}$	6.2×10^{-12}(h)	cQO_2: 6.7×10^{-12} $HOOQO_2$: 3.8×10^{-12} (prim) 8.0×10^{-14} (sec)(h)	4.1×10^{-12} (prim) 3.7×10^{-13} (sec)(h)	7.9×10^{-15} (tert)(h)
(14) RO_2 + $HOOQO_2$ or c-QO_2 → products	$cm^3\ s^{-1}$	2.0×10^{-12} (h)	1.2×10^{-12} (avg)(h,k)	1.3×10^{-12} (avg)(h,k)	1.1×10^{-13}(h)
(15) RO + O_2 → R=O + HO_2	$cm^3\ s^{-1}$	1×10^{-14} (f)	1×10^{-14} (f)	1×10^{-14} (f)	1×10^{-14} (f)
(16) $HOOQO_2$ or c-QO_2 + O_2 → product + HO_2	$cm^3\ s^{-1}$	1×10^{-14} (f)	1×10^{-14} (f)	1×10^{-14} (f)	1×10^{-14} (f)
(17) RO_2 + HO_2 → ROOH + O_2	$cm^3\ s^{-1}$	1×10^{-11} (f)	1×10^{-11} (f)	1×10^{-11} (f)	1×10^{-11} (f)
(18) $HOOQO_2$ + HO_2 → products	$cm^3\ s^{-1}$	1×10^{-11} (f)	1×10^{-11} (f)	1×10^{-11} (f)	1×10^{-11} (f)
(19) RO_2 → $HOOQO_2$ or c-QO_2	s^{-1}	3.5 (l)	0.3(l)	0.3(l)	250(l)
(20) RO_2 → walls	s^{-1}	3×10^{-3} (m)	3×10^{-3} (m)	3×10^{-3} (m)	3×10^{-3} (m)
(21) HOOQO2 or c-QO2 → products	s^{-1}	1.2×10^{-2} (i) / 1.0(l)	10 ((i), $HOOQO_2$) / 1 (c-QO_2)(l)	2.5 (i) / 1(l)	3.4×10^{-2} (i) / 25(l)
(22) unsaturated RH or RI + O → R′=O+R″-I	$cm^3\ s^{-1}$	2×10^{-10} (n)	2×10^{-10} (n)	2×10^{-10} (n)	2×10^{-10} (n)
(23) I + O_3 → IO + O_2	$cm^3\ s^{-1}$	1.4×10^{-12} (o)	1.4×10^{-12} (o)	1.4×10^{-12} (o)	/
(24) Br + O_3 → BrO + O_2	$cm^3\ s^{-1}$	/	/	/	1.2×10^{-12} (o)
(25) IO + RO_2 → RO + I + O_2 (25%)	$cm^3\ s^{-1}$	2×10^{-11} (p)	2×10^{-11} (p)	2×10^{-11} (p)	/
(26) BrO + RO_2 → RO + Br + O_2 (25%)	$cm^3\ s^{-1}$	/	/	/	6×10^{-12} (p)
(27) IO+ HO_2 → HO+ I + O_2	$cm^3\ s^{-1}$	8.4×10^{-11} (o)	8.4×10^{-11} (o)	8.4×10^{-11} (o)	/
(28) IO + O_3 → I + 2 O_2	$cm^3\ s^{-1}$	$< 1 \times 10^{-15}$ (o)	$< 1 \times 10^{-15}$ (o)	$< 1 \times 10^{-15}$ (o)	/
(29) Br + R-C=C-R′ →	$cm^3\ s^{-1}$	/	/	/	1×10^{-13} (q)

*measured experimentally; (a) estimated from σ(5-Bromo-2-methyl-2-pentene) = 1×10^{-20} cm^2;[1] (b) estimated from σ(O_2) = 2.5×10^{-25} cm^2;[1] (c) estimated assuming σ(RO_2) ~ σ(HO_2) = 1×10^{-19} cm^2;[1] (d) estimated from σ(t-butylOO-t-butyl) = 2.5×10^{-20} cm^2;[1] (e) estimated using σ(t-butylOOH) = 2.5×10^{-20} cm^2; (f) estimated from Ref.[2] for primary RO_2; (g) Ref[3]; (h) Ref.[4], where it is assumed that an β-OOR or β-OOH substituent group has a similar effect as a β-OH group, and ignoring the presence of a ring; (i) from the SAR of Ref.[5]; (k) Geometric average of the rates for the various $HOOQO_2$/c-QO_2 radicals; (l) adjusted to fit the observed kinetics (see text); (m) determined experimentally; (n) from Ref.[2] for CH_4; (o) from ref[6] ;.

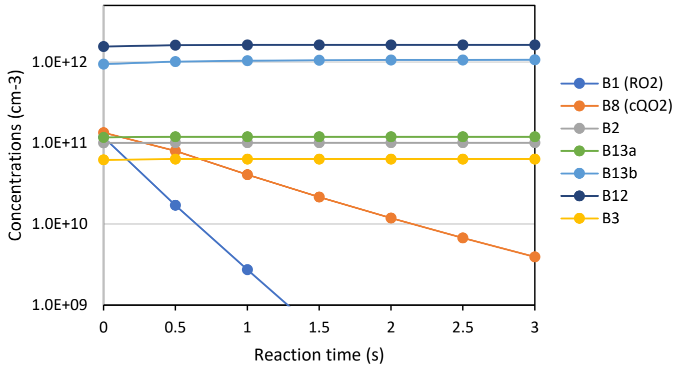

Fig. S4.1: Kinetic simulations for the 1-butenyl-O_2 system. B13a is the acrolein produced by the RO_2 self-reaction channel and B13b is that produced in the cyclisation channel.

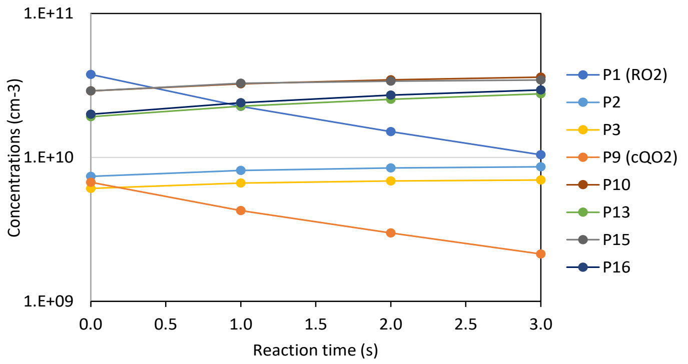

Fig. S4.2: Kinetic simulations for the 1-pentenyl-O_2 system.

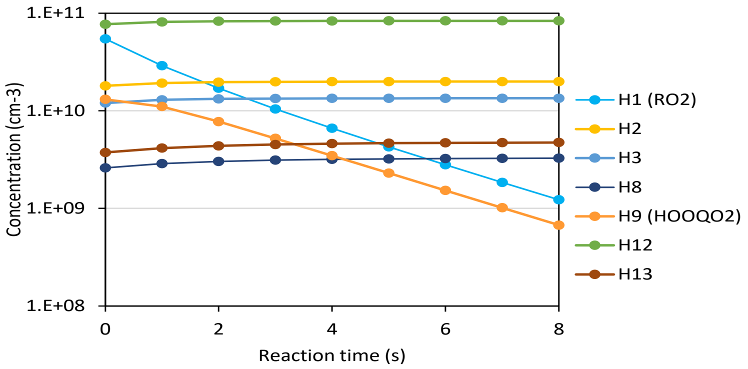

Fig. S4.3: Kinetic simulations for the 1-hexenyl-O_2 system.

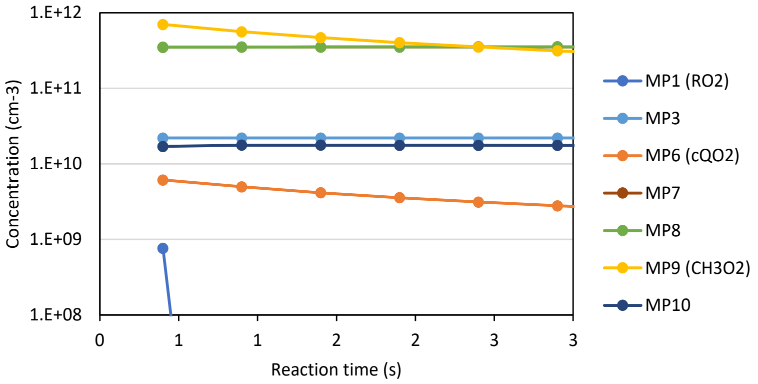

Fig. S4.4: Kinetic simulations for the 2-methyl-2-pentenyl-O_2 system (note that products MP7 and MP8 overlap).

Section S5. Mass spectra and main ions detected in the experiments

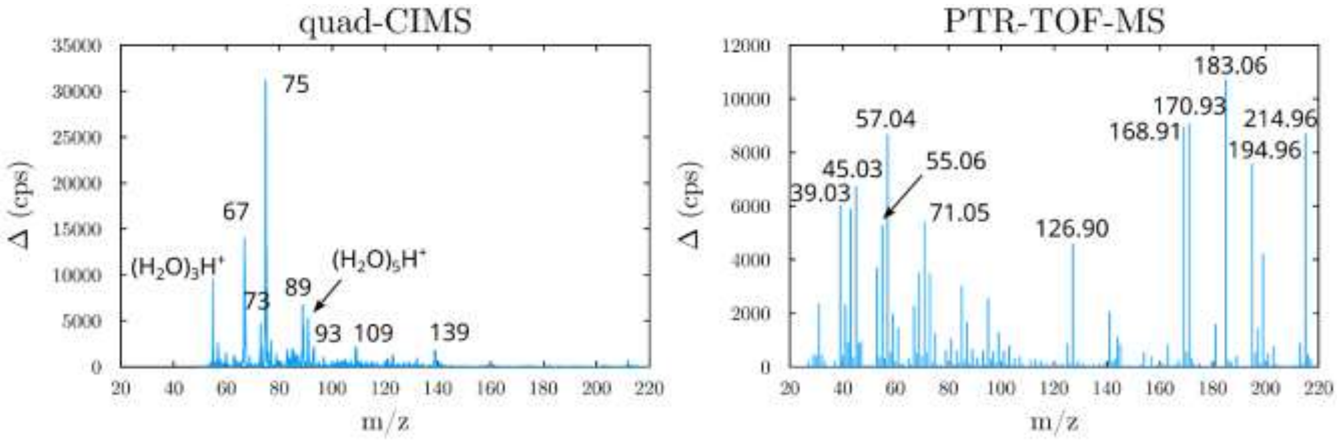

***Figure S5a**: Mass spectra for the 1-butenyl-O_2 system Left: CIMS analysis; Right: PTR-TOF-MS analysis*

***Table S5b**: List of the main ions (and intensities in Hz or cps) observed in the 1-butenyl-O_2 system.*

CIMS	*Ptr-tof-MS FUSION*	*Proposed compound or ion*	*Product # in mechanism*
57/75/93 (2600/31000/2200)	57/75 (22000/1000)	C_3H_4O-H^+ *m/z* 57<br>C_3H_4O-$(H_2O)H^+$ *m/z* 75<br>C_3H_4O acroleine 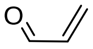	B13
67/85 (14000/1900)	31 (3200)	CH_3OOH-H^+ *m/z* 49<br>CH_3OOH-$(H_2O)H^+$ *m/z* 67<br>CH_3OOH-$(H_2O)_2H^+$ *m/z* 85<br>Detected as CH_3O^+, *m/z* 31 in PTR-tof-MS	Co-product B13
71/89 (700/6700)	71 (6100)	C_4H_6O-H^+, *m/z* 71<br>C_4H_6O-$(H_2O)H^+$, *m/z* 89<br>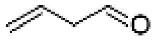	B3
73/91/109 (4000/5000/2100) *m/z* 73 and 91 overlap with ion clusters	73 (4200)<br>+ 55 (2600)	C_4H_8O-H^+, *m/z* 73<br>C_4H_8O-$(H_2O)H^+$, *m/z* 91<br>C_4H_8O-$(H_2O)_2H^+$, *m/z* 109<br>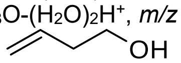<br>Partly dehydrates into C_4H_6-H^+ , *m/z* 55 in PTr-tof-MS	B2
63 (1150)	45 (7200)	acetaldehyde	?
103/121/139 (400/930/1800)	103 (1000)<br>+<br>69 (5000)	$C_4H_6O_3$-H^+, *m/z* 103<br>$C_4H_6O_3$-$(H_2O)H^+$, *m/z* 121<br>$C_4H_6O_3$-$(H_2O)_2H^+$, *m/z* 139<br>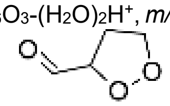<br>Partly detected as $C_4H_5O^+$, *m/z* 69 in PTR-tof-MS 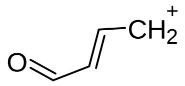	B12
	85 (3000)	$C_4H_4O_2$-H^+, *m/z* 85<br>Unidentified product or ion fragment	
83 (1800)		Unidentified product or ion fragment	
138/156/174 (total ~190)	/	$C_4H_7O_4(H_2O)H^+$, *m/z* 138<br>$C_4H_7O_4(H_2O)_2H^+$ *m/z* 156.1<br>$C_4H_7O_4(H_2O)_3H^+$ *m/z* 174.1<br>c-QO_2, 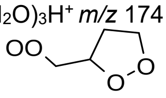	B8
106/124/142 (total ~130 Hz)	/	$C_4H_7O_2(H_2O)H^+$: 106.1<br>$C_4H_7O_2(H_2O)_2H^+$: 124.1<br>$C_4H_7O_2(H_2O)_3H^+$: 142.1<br>RO_2, 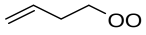	B1

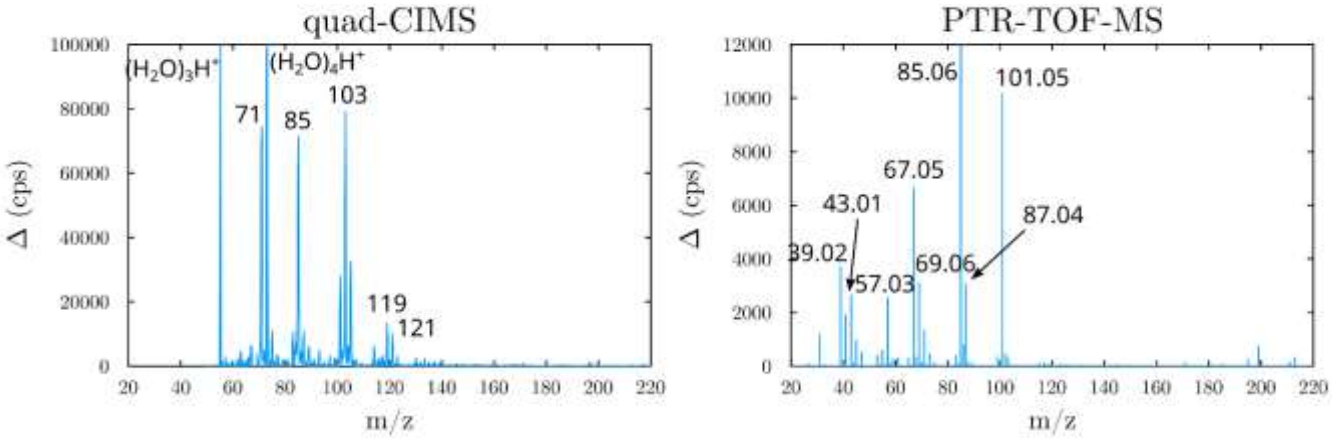

***Figure S5c**: Mass spectra for the 1-pentenyl-O_2 system. Left: CIMS analysis; Right: PTR-TOF-MS analysis*

***Table S5d**: List of the main ions identified (and intensities) in the 1-pentenyl-O_2 system.*

CIMS	*Ptr-tof-MS FUSION*	*Proposed compound or ion*	*Product # in mechanism*
85/103/121 (71000/79000/10000)	85 (19000)	C_5H_8O-H^+, *m/z* 85<br>C_5H_8O-$(H_2O)H^+$, *m/z* 103<br>C_5H_8O-$(H_2O)_2H^+$, *m/z* 121<br>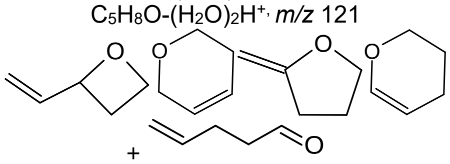	P3, P10a,P10b, P10c, P10d
87/105/123 (10800/32000/1500)	87 (3100)<br>+<br>69 (3100)	$C_5H_{10}O$-H^+, *m/z* 87<br>$C_5H_{10}O$-$(H_2O)H^+$, *m/z* 105<br>$C_5H_{10}O$-$(H_2O)_2H^+$, *m/z* 123<br>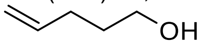<br>Partly dehydrates into $C_5H_8^+$, *m/z* 69 in PTR-tof-MS	P2
83/101/119 (10000/28000/13000)	83/101 (400/10000)	Fragmentation of ion $C_5H_8O_3$-H^+ *m/z* 117<br>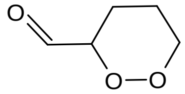<br>into $C_5H_7O^+$, *m/z* 83<br>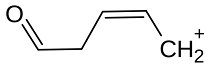	P13
66/84 (1900/7000)	/	CH_3O_2	
71/89/107 (74000/5300/2000)	71 (1300)	C_4H_6O-H^+, *m/z* 71<br>C_4H_6O-$(H_2O)H^+$, *m/z* 89<br>C_4H_6O-$(H_2O)_2H^+$, *m/z* 107<br>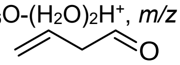	P15
67 (6200)	67 (6600)	C_5H_6-H^+, m/z 67<br>Dehydration of ion C_5H_8O-H^+<br>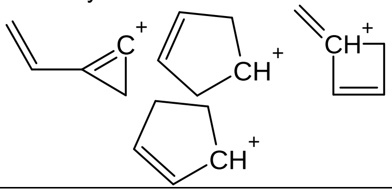	P10a, P10b, P10c, P10d
75/93 (10500/4900)	57 (2500)	C_3H_4O-H^+ *m/z* 57<br>C_3H_4O-$(H_2O)H^+$ *m/z* 75<br>C_3H_4O acrolein 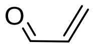	P16
63/81/99 (4300/2000/2500)	45 (950)	C_2H_5OOH-H^+, *m/z* 63<br>C_2H_5OOH-$(H_2O)H^+$, *m/z* 81<br>C_2H_5OOH-$(H_2O)_2H^+$, *m/z* 99<br>Detected at $C_2H_5O^+$, *m/z* 45 in PRT-tof-MS	Co-product P16
	43 (2600)	Unidentified product or ion fragment	
97 (3000)		Unidentified product or ion fragment	
120/138/146 (total ~ 170)	/	$C_5H_9O_2(H_2O)H^+$ = 120.1<br>$C_5H_9O_2(H_2O)_2H^+$ = 138.1<br>$C_5H_9O_2(H_2O)_3H^+$ = 156.1<br>RO_2, 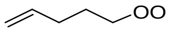	P1

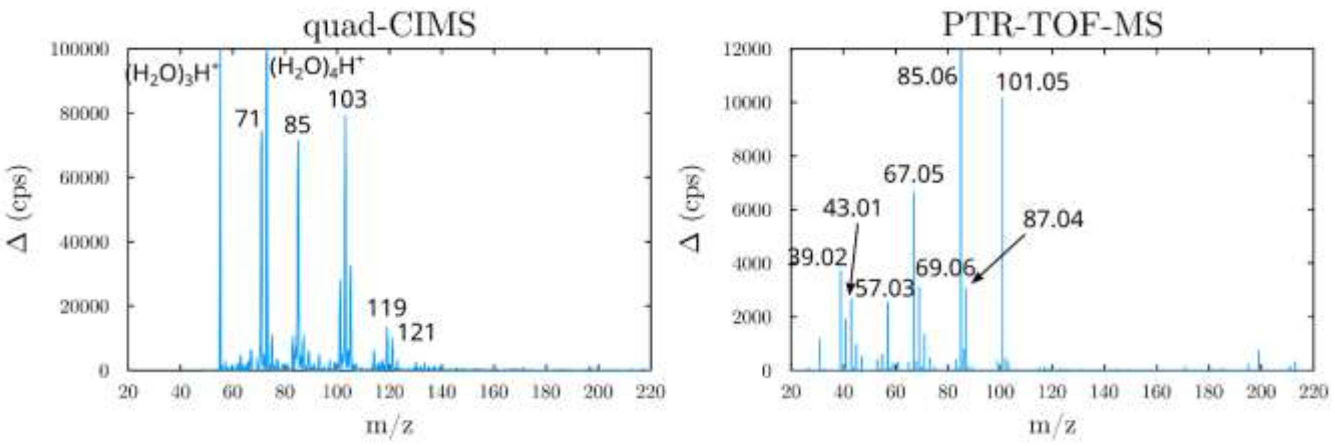

Figure S5e: *Mass spectra for the 1-hexenyl-O_2 system. Left: CIMS analysis; Right: PTR-TOF-MS analysis*

***Table S5f**: List of the main ions identified in the 1-hexenyl-O_2 system.*

CIMS	*Ptr-tof-MS FUSION*	*Proposed compound or ion*	*Product # in mechanism*
81 (21000)	81 (26500)	$C_6H_8H^+$ 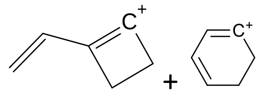 Dehydration of ion $C_6H_{10}O\text{-}H^+$	H8
99/117 (4600/8700)	99 (9600)	$C_6H_{10}O\text{-}H^+$ 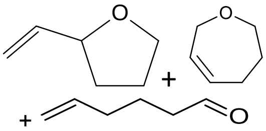	H8, H3
63 (1600)	45 (9500)	Acetaldehyde	
97 (4000)	97 (5300)	$C_6H_8O\text{-}H^+$ unidentfied compound	
75 (1300)	57 (6600)	$C_3H_4O\text{-}H^+$, acrolein	H13
85/103 (3600/600)	85 (3800)	$C_5H_8O\text{-}H^+$, 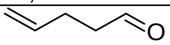	H12
101/119 (1500/3600)	82 /101 (3300/2700)	$C_5H_8O_2$ $-H^+$, or 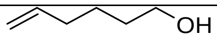	H2
67/85 (1600/3600)	67 (3800) +31 (6200)	$C_5H_6\text{-}H^+$ or H_3COOH	
59 (600)	59 (4700)	$C_3H_6O\text{-}H^+$ acetone	
79 (600)	79 (4000)	$C_6H_6\text{-}H^+$ unidentified compound	Fragment ion H17a,H17b, H17c, H17d ?
69 (1500)	69 (4000)	$C_5H_8\text{-}H^+$ unidentified compound	Fragment ion H17a,H17b, H17c, H17d ?
134/152/170 (total ~ 760)		$C_6H_{11}O_2(H_2O)H^+$ = 134.1 $C_6H_{11}O_2(H_2O)_2H^+$ = 152.1 $C_6H_{11}O_2(HO_2)_3H^+$ = 170.1 RO_2 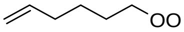	H1

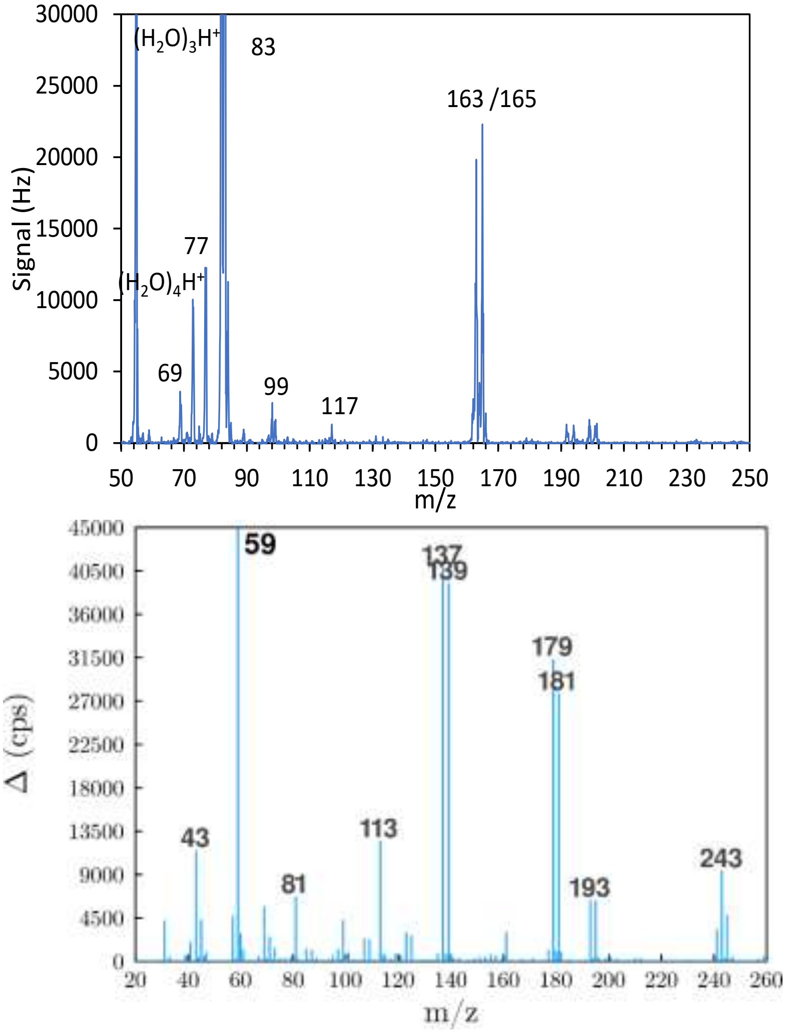

Figure S5g: *Mass spectra for the 2-methyl-2-pentenyl-O_2 system. Top: CIMS analysis; Bottom: PTR-TOF-MS analysis*

***Table S5h**: List of the main ions identified in the 2-methyl-2-pentenyl-O_2 system.*

CIMS	*Ptr-tof-MS FUSION*	*Proposed compound or ion*	*Product # in mechanism*
83 (82000)	/	In from brominated precursor + 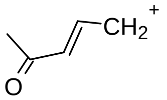 $C_5H_7O^+$ fragmentation of ion $C_5H_8O_3$-H+, 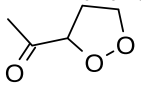 *m/z* 117	MP8
59/77 (900/12000)	59 (108000)	C_3H_6O-H^+ acetone	MP10
163/165 (20000/20000)	/	brominated precursor	
/	113 (12500)	unidentified compound	
66/84 (50/11000)	/	CH_3O_2	MP9
69 (3500)	69 (5600)	C_5H_8-H^+ ion fragment	
57/75 (700/1100)	57 (4600)	C_3H_4O–H+, acrolein	MP7
81 (900)	81 (6700)	C_6H_8-H^+ ion fragment	
99/117/135 (1600/1300/250)	99 (4300)	$C_6H_{10}O$-H^+ *m/z* 99 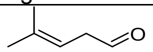	MP3
	45 (4300)	?	
67/85 (350/1500)	31 (4200)	H_3COOH	

Section S6. Mechanistic considerations

In this section we examine some possible mechanisms that might be useful to explain to the observations. In most cases we rely on theoretical calculations and/or available literature data, but this section remain highly speculative and should not be considered anything other than exploratory.

Decomposition of c-QO2 and formation of acrolein

The experiments with 1-butenyl-O_2 and 2-Me-2-pentenyl-O_2 are both predicted to proceed by cyclisation to a five-membered cycloperoxide (shown in Scheme S6.1 for 1-butenyl-O_2).

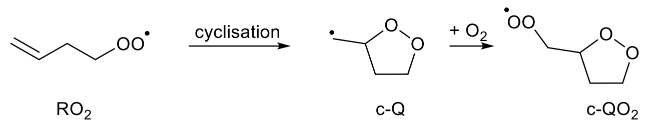

Scheme S6.1

The product ions observed experimentally for these RO_2 suggest that the c-QO_2 formed from RO_2 ring closure might decompose into smaller products, such as acrolein and acetone. This is mostly based on the very intense signals observed for acrolein with 1-butenyl-O_2 (22000 – 31000 cps) and for acetone with 2-Me-2-pentenyl-O_2 (≥ 12000 cps). Assuming typical detection sensitivities of ~ 20000 cps/ppb for acrolein and 40000 cps/ppb for acetone these observed ion intensities correspond to ~ 1 – 1.5 ppb of acrolein from 1-butenyl-O_2 (thus a yield of ~ 33 - 50 %) and 0.3 ppb acetone from 2-Me-2-pentenyl-O_2 (thus a yield of ~ 100 %). For 2-pentenyl-O_2, only about half of the reaction flux is predicted to go through 6-membered cyclisation (see Table 1), but even for this radical the acrolein signal is much larger (~10000 cps thus ~ 0.5 ppb and a yield of 25 %) than with 1-hexenyl-O_2, which is not expected to undergo cyclisation directly. For 1-hexenyl-O_2, the much smaller acrolein signals observed (1300 cps with the CIMS, thus 0.065 ppb and 0,65 % of the initial RO_2) could result from the cyclisation of the dominant $HOOQO_2$, mostly to a 6-membered cyloperoxide HOO-c-QO_2 (see Fig. 5). In addition, the direct observation of the c-QO_2 with 1-butenyl-O_2 and of intense ion signals that can be attributed to the carbonyl product of the c-QO_2 with the other RO_2, further support the fact that these c-QO_2 are indeed formed. Overall, this data suggests that the cyclic peroxide c-QO_2 formed from cyclisation readily dissociate, forming acrolein from the cyclic moiety, while the exo-cyclic moiety is converted to a carbonyl, e.g. through an alkoxy radical decomposition separating it from the ring (e.g. acetone with 2-methyl-2-pentenyl-O_2).

The mechanism explaining such decomposition is unclear. Vereecken et al.[7] found that the ring closure process is near-energy neutral, such that no dominant chemically activated breaking of the weaker O–O bond (BDE 40-45 kcal/mol) in the c-Q alkyl radical is expected. Relative to collisional energy loss, the O_2 addition itself is moderately slow under atmospheric conditions, ~5×10^7 s^{-1},[8] further corroborating the thermalization of the c-Q. The O_2 addition forming c-QO_2 is exothermic by 30-35 kcal/mol, but our theoretical calculations show that O–O bond breaking in c-QO_2, forming a tri-radical, is endothermic by about 30 kcal/mol even accounting for the release of ring strain in the 5-membered ring (Fig. S6.2).

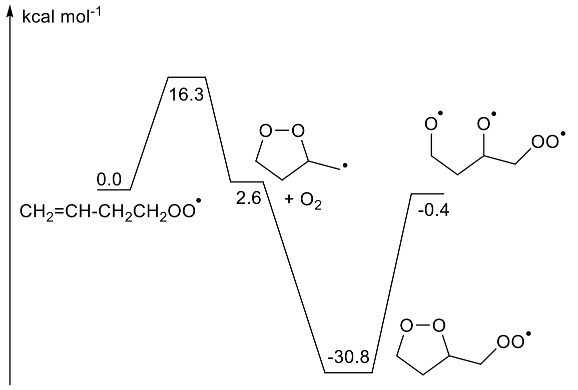

Figure S6.2.

Hence, the O_2 addition energy release is barely enough to break the weakest bond, making bond fission in the c-QO_2 negligible against thermalization. Additionally, Vereecken et al. [7] showed that the c-QO_2 radical as shown

above does not have fast H-migration reactions, the fastest channel being a 1,6-H-migration across the 5-membered ring with k(298 K) = $1×10^{-2}$ s^{-1}. Hence, further autoxidation of the c-QO_2 does not seem to provide an accessible pathway to high yields of acrolein formation.

We also examined the fate of the c-QO alkoxy radical, formed from c-QO_2 after reaction with NO or RO_2, as a potential source of acrolein. The steps are shown in Scheme S6.3 for the c-QO_2 formed from 2-Me-2-pentenyl-O_2.

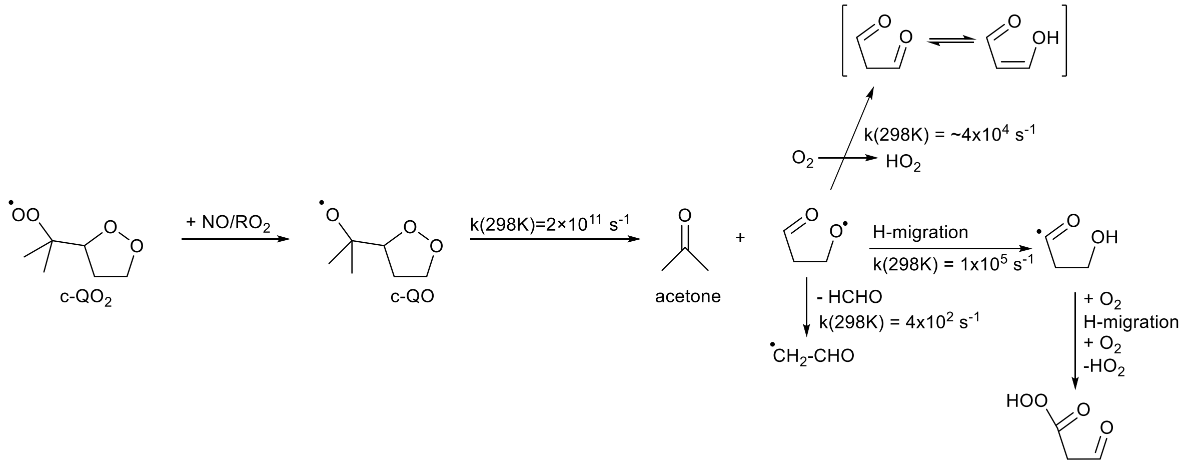

Scheme S6.3

Decomposition of such a c-QO is indeed expected to readily form the observed acetone, with a rate ≥10^{11} s^{-1}.[9, 10] The cycloperoxide fragment has an α-OOR alkyl radical site, which are known to be unstable and readily breaks the O–O bond, forming a carbonyl group and an alkoxy radical.[11] The alkoxy radical can undergo an aldehydic 1,4-H-migration (k(298K) = $1×10^5$ s^{-1}), elimination of CH_2O (k(298K) = $4.1×10^2$ s^{-1}[9]), or reaction with O_2 (k(298K) ~ $4×10^4$ s^{-1} for 0.2 atm O_2) forming O=$CHCH_2CH$=O. None of these products show a facile route to acrolein or any of its isomers, even if we account for a potentially high internal energy content. It is unclear at this time how the predicted products respond to ionization by H^+/$H^+(H_2O)_n$.

At this point we should mention that the proposed mechanisms have some pathways leading to acrolein formation through well-known channels in peroxy and alkoxy chemistry, such as the decomposition sequence shown in Scheme S6.4 starting at 1-hexenyl-O_2.

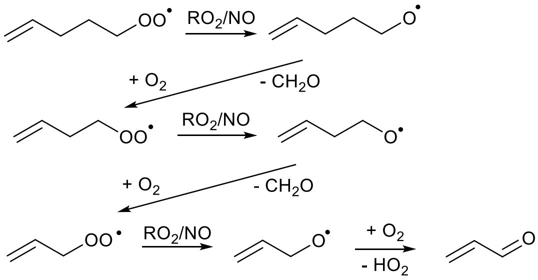

Scheme S6.4.

While this sequence has some viability starting at 1-butenyl-O_2, where CH_2O elimination is the fastest channel, the chemistry of unsaturated alkoxy radicals with longer carbon chains are dominated by allylic H-migration and ring closure reactions, none of which seem to lead to acrolein formation. As acrolein is observed for all unsaturated RO_2 reacting through ring closure, traditional RO_2+RO_2/HO_2/NO chemistry as shown above is not tenable as the main acrolein-forming mechanism for all RO_2 studied here. Additionally, our modeling shows that the self- and cross-reactions of the RO_2 only contribute for a small fraction.

Formation of unsaturated cyclo-ethers, carbonyls, alcohols, or epoxides

For all the RO_2 radicals studied in this work, the mass spectra displayed an ion peak corresponding to a stable product with a *m/z* at 17 mass units below that of the RO_2, along with the corresponding water/proton clusters in the CIMS: *m/z* 71/89 for butenyl-O_2, *m/z* 85/103 for 1-pentenyl-O_2, and *m/z* 99/117 both for hexenyl-O_2, and 2-

methyl-2-pentenyl-O_2. These masses correspond to the main carbonyl compounds expected from the self-reactions of the RO_2 but also to other isomers. For instance, for 1-pentenyl-O_2, they correspond to compounds with the sum formula C_5H_8O such as in Fig. S6.5.

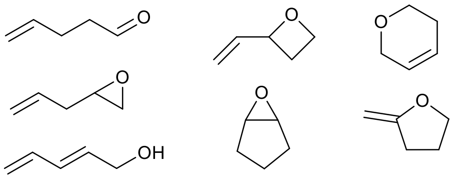

Figure S6.5.

However, these signals were much more intense in the 1-pentenyl-O_2 and 1-hexenyl-O_2 systems than in the butenyl-O_2 and 2-methyl-2-pentenyl-O_2 systems (9000 – 80000 cps vs 4000 – 6500 cps), suggesting that they resulted from other isomers than the linear carbonyl compounds expected from the RO_2 self-reactions (see discussion below). Furthermore, for 1-pentenyl-O_2 and 1-hexenyl-O_2, which react near-exclusively by allylic H-migration, intense peaks were observed at *m/z* that could potentially be attributed to dehydrated ions resulting from some of the cyclic isomers presented in Schemes S6.14 and S6.16: *m/z* 67, 6500 cps for 1-pentenyl-O_2, and *m/z* 81, 25000 cps for 1-hexenyl-O_2. These products were not present with butenyl-O_2 and 2-methyl-2-pentenyl-O_2, which react mostly by cyclisation, thus were attributed to the product channels related to the allylic H-migration. Assuming also a typical detection sensitivity of 30000 cps/ppb for these products, the observed intensities corresponded to ~ 1 ppb with 1-pentenyl-O_2 (60000 cps in average) thus a 66 % yield and 1.2 ppb with 1-hexenyl-O_2 (~ 35000 cps) and a ~ 55 % yield. Below, we discuss some possible mechanisms for the formation of products of this mass, illustrating the pathways using 1-pentenyl-O_2.

Mechanism A: carbonyl channel in primary RO_2+RO_2

As explained above, possible candidates for the intense ions at *m/z* 85/103 for 1-pentenyl-O_2 and *m/z* 99/117 for 1-hexenyl-O_2 are the carbonyl products formed from their self-reactions, as illustrated in Scheme S6.6.

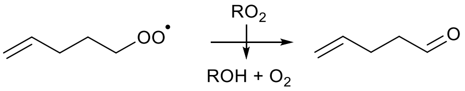

Scheme S6.6.

However, for the bimolecular RO_2+RO_2 reactions our modeling suggests that the pseudo-first order loss rate for these RO_2 by self-reaction would be ~ 0.17 s^{-1} for 1-pentenyl-O_2 and 0.18 s^{-1} for 1-hexenyl-O_2 (albeit with large uncertainties on the RO_2 concentrations indicated in Table S1). This represented only ~ 45 - 60 % of the measured overall losses for these radicals (~ 0.3 s^{-1}), the latter being largely attributed to their unimolecular processes. Assuming a similar sensitivity for the primary RO_2 and the unknown product, it would require an increase of a factor 1.5 – 2.5 of the rate of primary RO_2+RO_2 compared to the recommendation by Jenkin et al.[4] to account for the observed RO_2 decays but factors 4.5 – 7.5 to allow sufficient alkenone formation. In addition, these carbonyl products would not explain the observation of the intense ions corresponding to the “dehydrated” cyclic ethers.

Mechanism B: cycloether+OH formation from HOOQ

Other potential products accounting for the observed intense ions might be the direct formation of a cyclic ether with OH elimination from the HOOQ intermediate, as illustrated for the 1-pentenyl-O_2 system in Scheme S6.7.

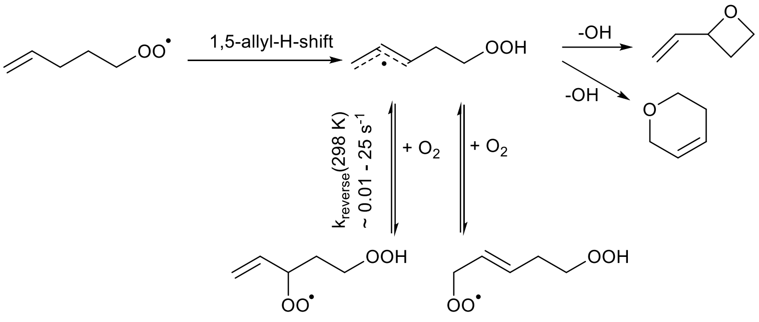

Scheme S6.7.

This process is well-known in combustion for hydroperoxy-substituted alkyl radicals,[12-14] with barrier as low as 10 kcal mol^{-1}. To our knowledge, no literature data exists on the rate of (non-epoxide) cyclic ether + OH formation for allylic radicals. Our theoretical calculations predict barriers in excess of 25 kcal mol^{-1} for the 5-OOH-2-butenyl allylic radical, with rate coefficients of k(298 K) ≤ 10^{-6} s^{-1} (see Table S7.2), in agreement with the low rates for epoxide formation predicted by Møller et al.[15] This ring closure occurs in competition with the reversible O_2 addition on the allylic radical site, and through that with the loss processes of the $HOOQO_2$ radicals (see Scheme S6.7). All these competing processes occur with rates several orders of magnitude faster than the cyclic ether + OH channel, making the latter likely a negligible channel (see Novelli et al. [16] for estimated rates for O_2 addition and re-elimination from allylic RO_2 radicals).

Mechanism C: cyclisation of primary alkoxy radicals, followed by HO_2 elimination

Other potential pathways for the formation of cyclic ethers could be the cyclisation of (neutral) alkoxy radicals followed by HO_2 formation, either concertedly or in an H-shift/elimination sequence as illustrated for the 1-pentenyl-O_2 system in Scheme S6.8:

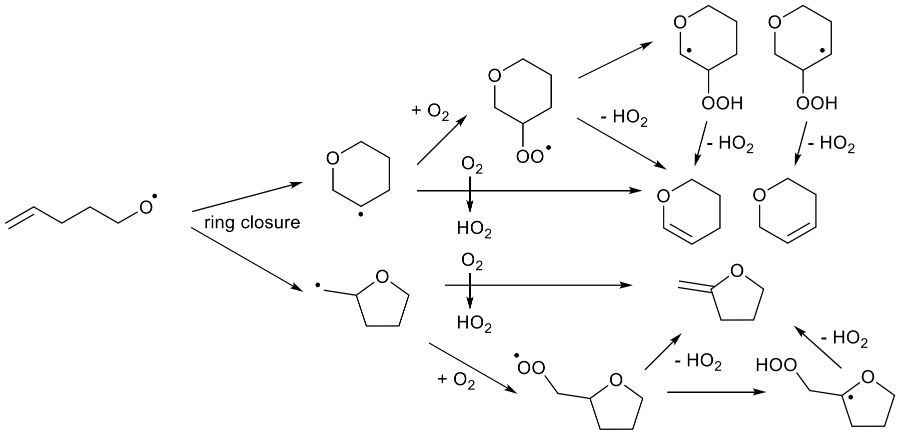

Scheme S6.8.

The alkoxy radical is the main product from the primary RO_2 in its reactions with RO_2 or NO. The potential energy surface of this reaction system has been calculated theoretically:

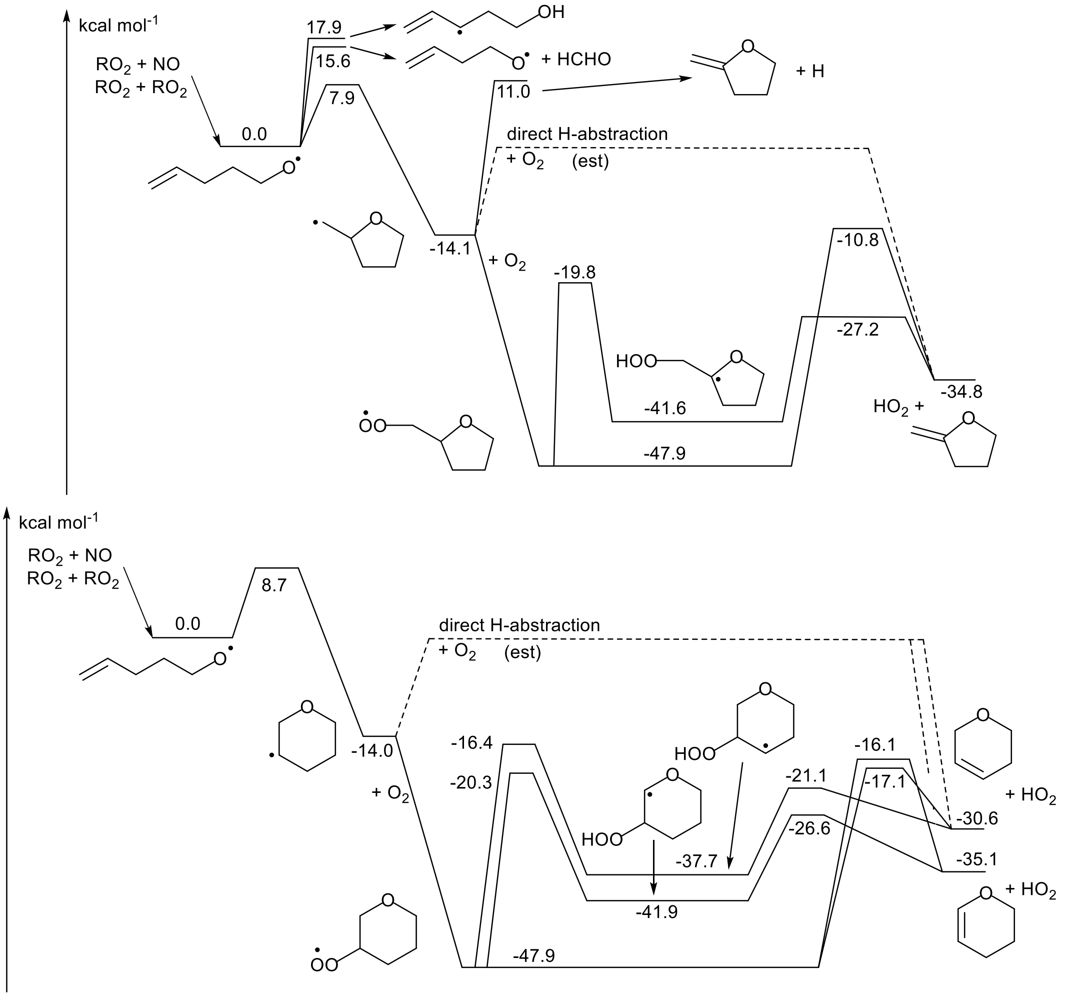

Figure S6.9:

For regular alkyl radicals, O_2 addition is not sufficiently exothermic to allow sizable amounts of HO_2 + alkene formation at atmospheric temperature. Though it is often overwhelmed by more facile RO_2 H-migration reactions, HO_2 + alkene formation is, however, a pathway that is known to occur at combustion temperatures (see e.g. Ref.[17]), indicating that additional internal energy enhances its relative importance. Its main formation pathway is an addition/H-migration/HO_2-elimination mechanism, as direct H-abstraction by O_2 has a high barrier (see e.g. Ref.[18]), while concerted HO_2 elimination is calculated here to have slightly higher barriers. For the cycloether peroxy radicals studied here, competing H-migrations are less favorable due to the ring structure.[7] Table S7.2 shows rate coefficients for the thermal elementary reactions in the mechanisms, indicating that the reactions are too slow to compete with regular loss processes. However, the cyclo-ether alkyl radical is chemically activated from the alkoxy ring closure reaction, and the alkoxy radical itself may already be formed with an enhanced energy content from the RO_2 + RO_2 or RO_2 + NO formation reaction. If this increased internal energy is (partly) carried over to the cyclo-RO_2, this could enhance the formation of cyclic unsaturated ethers + HO_2 to measurable levels. Even for thermal alkoxy radicals, the ring closure reaction is the dominant loss process (see Table S7.2), dominating other unimolecular reactions and reaction with O_2 ($k \sim 4\times10^4$ s^{-1} at 0.2 atm O_2).[19] The PES shown above are not complete, as they don't include potential competing reactions from other RO_2 H-migrations, or epoxy+OH formation; a full Master Equation analysis quantifying the effect of chemical activation is not in the scope of the present work. A critical parameter will be the rate of O_2 addition, which is not overly fast (~ 10^7 s^{-1} at 0.2 atm O_2), leaving time for collisional energy loss in the cycloether-peroxyl intermediate. It should also be noted that, for the unsaturated RO_2 studied in this work, this mechanism is most viable for 1-pentenyl-O_2 (see the discussion of the annotated mechanism for 1-pentenyl-O_2 below for some corroborating evidence). This process would form isomers of the P10 product, where the formation of cyclic ethers was strongly supported experimentally by the observation of intense ions in the 1-pentenyl-O_2 and 1-hexenyl-O_2 systems, corresponding to dehydrated ions from these ethers. For 1-butenyl-O_2, the alkoxy ring closure yields a strained 4-membered ring and is energetically unfavorable; for 1-hexenyl-O_2 the alkoxy ring closure forming 6 and 7-membered rings is entropically less favorable and is likely

outperformed by the fast allylic 1,5-H-migration (see section S6); for 2-Me-2-pentenyl-O_2, the ring closure in the primary RO_2 is very fast such that only a negligible fraction of the primary RO_2 can react with RO_2/NO.

Mechanism D: cyclisation of hydroperoxyalkoxy radical HOOQO, followed by HO_2 elimination

Cyclic ether formation by ring closure in alkoxy radicals formed from RO_2 + RO_2 or RO_2 + NO reactions can also occur for some of the $HOOQO_2$ intermediates; as an example we show the following simplified potential energy surface for one of the $HOOQO_2$ intermediates from 1-pentenyl-O_2:

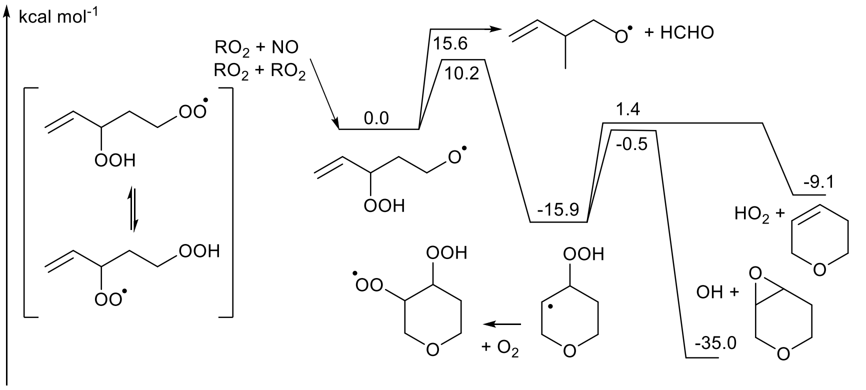

Figure S6.10:

The alkoxy ring closure reaction directly forms a β-OOH cycloalkyl radical that can undergo HO_2 elimination to form an unsaturated cyclo-ether with the searched-for mass. The main competition channel for HO_2 elimination is epoxide formation, which for thermalized linear β-OOH alkyl radicals with alkyl substituents was theoretically calculated by Møller et al.[15] to be 6 times faster than HO_2 elimination, with both of these channels several orders of magnitude slower than O_2 addition. For the present case, we must however consider the impact of the ring structure, the enhanced energy content imparted by the alkoxy ring closure, and the difference in entropy for the two loss processes. For thermal reaction we find that epoxidation is the fastest reaction channel, in agreement with Møller et al.[15] HO_2 elimination, however, is entropically more favorable, and gains in importance at higher temperatures or internal energies, such that cyclic-ether formation will gain in importance at the energies afforded by the exothermic ring closure; a full Master Equation analysis quantifying the effect of chemical activation is not in the scope of the present work.

The alkoxy ring closure reaction starting the reaction chain shown above faces competition from other unimolecular reaction channels (not shown), such as the formation of a 5-membered cycloether, fast H-migration reactions, and (slower) decomposition reactions. The reaction systems studied in this work have a multitude of unsaturated hydroperoxide-alkenylperoxy radicals that could undergo analogous complex alkoxy radical chemistry after reaction with RO_2 or NO. Hence, quantifying the yield of the hydroperoxide-cycloalkyl structure depicted above, or the yield of cycloethers in general is a complex undertaking and is outside the scope of the present study.

Mechanism E: unknown subsequent chemistry of $(HOO)_2QO_2$

We should consider that a product may be formed from the intermediates formed from the $HOOQO_2$ intermediate. These latter radicals are predicted to undergo mostly ring closure reactions with, depending on the addition site of the O_2, a rate of 0.16 or 48 s^{-1} for the 1-pentenyl-O_2 system. The most likely candidate for further autoxidation is the $HOOQO_2$ intermediate formed after O_2 addition on the inner C-atom of the HOOQ radicals, as illustrated in scheme S6.11:

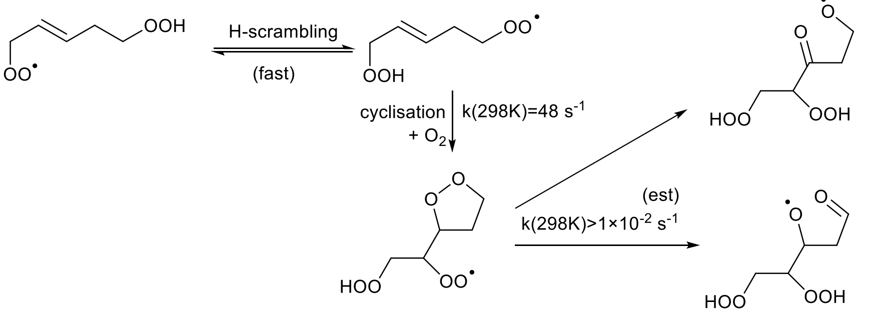

Scheme S6.11.

The subsequent chemistry of the resulting $(HOO)_2QO_2$ radicals is very complex with a highly branched mechanism, and theoretical calculations are expensive due to the large number of oxygen atoms. As such, it is unclear whether the target products could be formed. Similarly, we can't exclude that alkoxy radical intermediates formed from the reaction of RO_2, $HOOQO_2$, or $(HOO)_2QO_2$ intermediates with other RO_2 radicals could lead ultimately to products of the required mass; again, this chemistry is highly complex and no prominent direct pathway to such a product is obvious. A full analysis of this continued chemistry is outside the scope of this work. Still, one should consider that oxygen atoms, once attached to the backbone, are hard to remove again in subsequent reactions, such that forming a product with a stoichiometry of C_5H_8O for the 1-pentenyl-O_2 system, and analogously low-oxygenated products for the other systems, seems unlikely from the higher-oxygenated intermediates.

Mechanism F: Chemistry induced by protonation in the ionization chamber

The chemical ionization used in this work, based on $(H_2O)_nH^+$ clusters, is particularly soft and tends not to lead to fragmentation. At the same time, the unsaturated oxygenated radicals studied here may allow for chemistry that is not accessible in saturated species, and that may be enhanced in kationic form.

To probe the possibility of ionization-induced chemistry, we examined the fate of the allylic HOOQ intermediates similar to mechanism B, but upon addition of an H+ atom, probing reactions that might occur in the ionization cell based on $(H_2O)_nH^+$ clusters. Our preliminary calculations indicate that the proton adds to the –OOH moiety, rearranging without energy barrier to a H_2O moiety and forming a $[CH_2{=}CH\text{-}C^{\bullet}H\text{-}CH_2\text{-}CH_2O^{\bullet}]^+$ allyl-alkoxy biradical kation complexed with the H_2O. This radical readily cyclizes, forming the [cyclic ether + $H_2O]^+$ complex with a low barrier of about 6 kcal mol^{-1} ((see Scheme S6.12 top) These calculations are likely not a very good representation for the impact of $(H_2O)_nH^+$ clusters on the HOOQ intermediates, and the chemistry is expected to be significantly more complex. Still, they do suggest that the ionization could facilitate formation of the cyclic ether products in the ionization chamber. Overall, though, it seems unlikely that chemistry of the HOOQ in the ionization chamber is the main source of the prominent peak, as the HOOQ are lost by O_2 addition, H-scrambling and cyclization reactions with effective rates ≥ 1 s^{-1}, such that the concentration of free HOOQ radical reaching the ionization chamber is likely not very high.

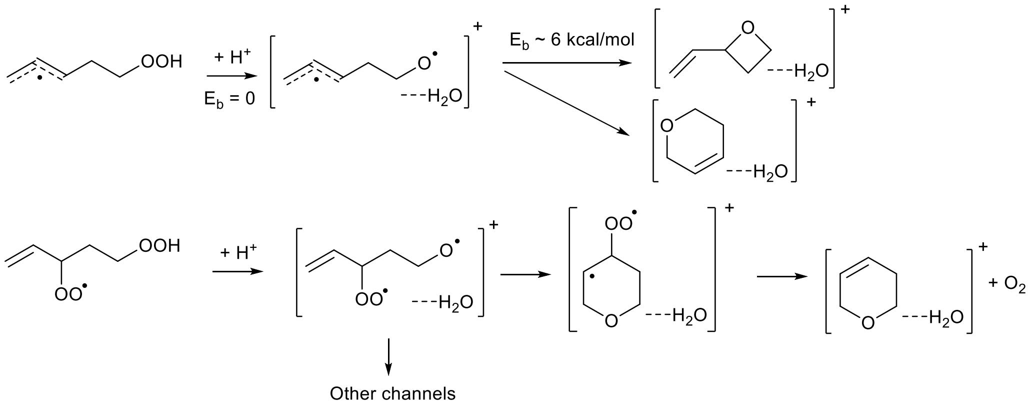

Scheme S6.12.

Another conceivable mechanism for C5H8O cyclic ether formation would be upon protonation of a $HOOQO_2$, which we illustrate here by a re-arrangement similar to mechanism D (see Scheme S6.12 bottom). This would lead to a β-OO$^{\bullet}$ alkyl radical, which would readily eliminate O_2 to form the double bond. We have not performed theoretical calculations on this mechanism, but assume the barriers are as low as for protonated mechanism B discussed above.

If the H^+-induced fragmentation of –OOH groups to an alkoxy group and H_2O is occurring more generally, the systems studied here have a potential of creating measurement artefacts owing to ion chemistry driven by the alkoxy chemistry formed from any –OOH group. As alkoxy chemistry is generally much faster than RO_2 chemistry, this could lead to high apparent yields of the products. As long as the products do not dissociate, however, the mass of the ion cluster remains the same, and would be indistinguishable from the original product. This makes assessing the importance of kationic chemistry very hard.

Formation of alkylhydroperoxides, CH_3OOH and C_2H_5OOH

The formation of small C1 and C2 compounds can have many sources in experiments such as described in this paper. Still, the experiments on 1-butenyl-O_2 and 1-propenyl-O_2 show masses equivalent to CH_3OOH and CH_3CH_2OOH, respectively, so in the interest of completeness it is worth to briefly examine potential formation pathways. Neither of the initiating compounds start out with a methyl group. Formation of a methyl group by H-migration to a R-$C^{\bullet}H_2$ radical would have to compete against addition of O_2 ($k \sim 5\times10^{7}$ s^{-1}), while aliphatic H-migration have theoretical and experimental rate coefficients $\leq 10^{4}$ s^{-1} at room temperature (e.g. Davis et al.[20, 21]). Even accounting for more mobile H-atoms (e.g. aldehydic, allylic or hydroperoxidic H-atoms), formation of competitive yields of methyl groups seems unlikely through such an H-migration pathway. We can't exclude that H-migration is more facile in the ionization chamber, where complexes with $(H_2O)_n$-H_3O^+ may have access to mobile protons, but earlier experiments on saturated RO_2[22] have not shown this to be an important channel.

The diols $HOCH_2OH$ and $HOCH_2CH_2OH$ are alternative isomers to the alkylhydroperoxides discussed above that do not require the formation of a methyl moiety. Diols are known to be formed from hydration of aldehydes [e.g; Winkelman et al.[23]], and are even the dominant forms in aqueous solutions of aldehydes. However, the gas phase oxidation schemes do not provide any viable pathways to (gemini-)diols and, while water is present in the ionization chamber, the experimental setup is not known to show a clear peak for the equivalent diol when measuring aldehydes.

Finally, we surmise that the alkylhydroperoxides are co-products of acrolein, which we assigned to a hitherto unknown decomposition channel of the cycloperoxide alkylperoxy radicals formed from cyclisation of unsaturated RO_2 (see section S6). We currently have no proposal how this re-arrangement/dissociation would take place.

As no obvious routes to either C1/C2 alkylhydroperoxides or diols were identified, the chemical speciation and the formation pathways of the molecules generating these mass peaks remains unclear at this time.

1-butenyl-O_2 annotated mechanism

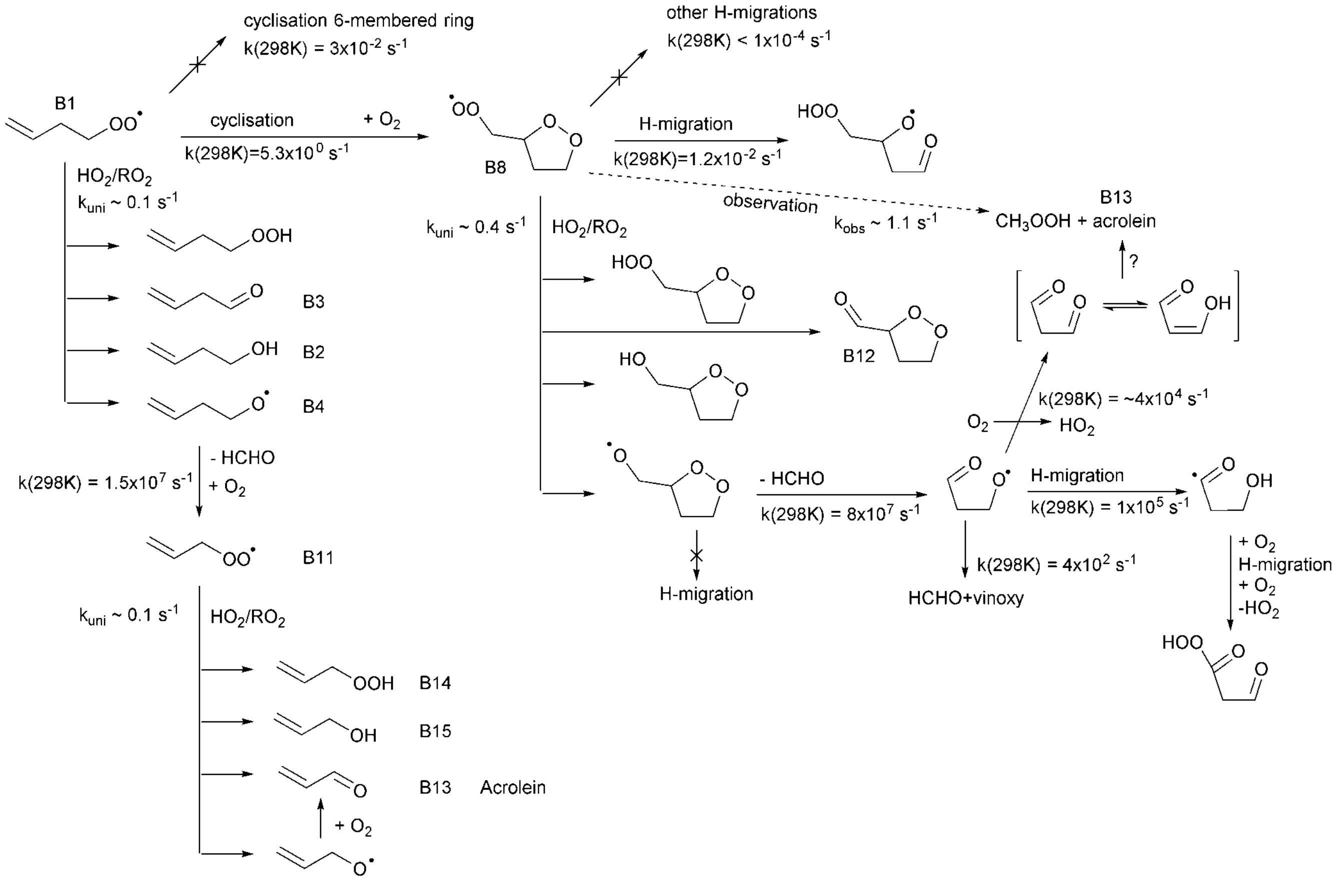

Figure S6.13: Extended mechanism for the oxidation of 1-butenyl-O_2 radicals

The rate coefficients for unimolecular reactions of 1-butenyl-O_2 radicals (B1) were calculated in this work (see Table S7.1), where the dominant route is cyclisation to a 5-membered peroxide ring (alkyl radical B5. Model calculations estimate the bimolecular loss by reactions with HO_2/RO_2 at a pseudo-first order rate coefficient of about 0.1 s^{-1}. These latter reactions lead to directly to some observed products and can lead to an alkoxy radical B4. SAR[9] predict that formaldehyde elimination is the dominant fate of this radical, forming an allyl-peroxy radical that has no viable unimolecular loss processes and thus reacts with HO_2/RO_2 to form a number of stable products, or an alkoxy radical that will react with O_2 to form acrolein. Acrolein formation through this sequence of reactions is slow due to the need of two reactions with RO_2, and is unlikely to have a high yield due to the competing formation of alcohols, ketones, hydroperoxides, and the cyclisation route in B1.

The cycloperoxide alkyl product formed from B1 will add O_2,[7] forming peroxy radical B8. The study by Vereecken et al.[7] showed no fast unimolecular loss processes for B8, while reactions with RO_2 and HO_2 are modelled to contribute a loss of ~0.43 s^{-1}, which is also the dominant loss in the theoretically predicted and modelled loss processes. The observed time evolution for B8, requires an additional loss process of ~0.9 s^{-1}. The expected chemistry does not show formation pathways to acrolein in yields that are sufficient to explain the peak heights observed in the mass spectra. We refer to the discussion in section S6 regarding acrolein formation.

1-pentenyl-O2 annotated mechanism

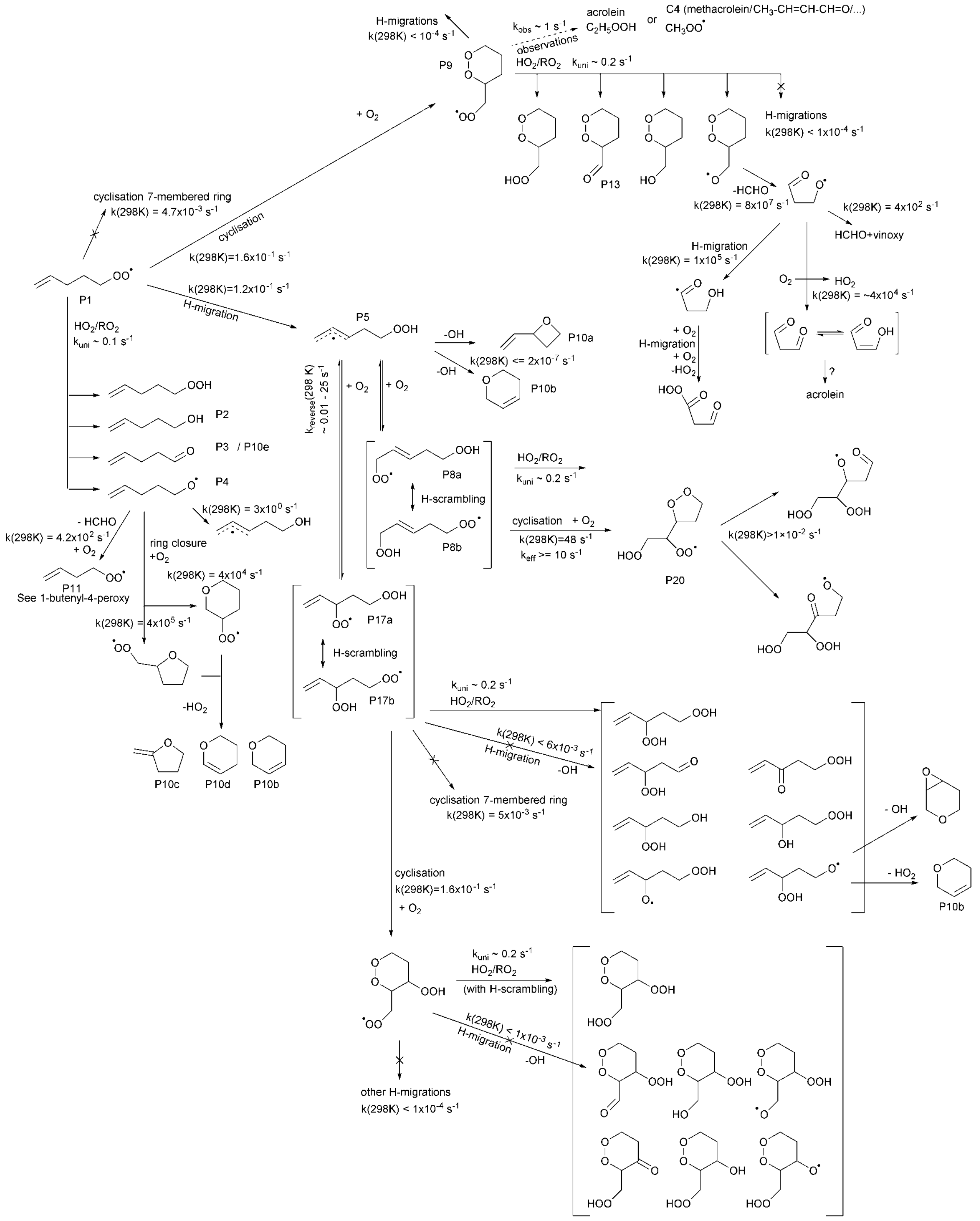

Figure S6.14: Extended mechanism for the oxidation of 1-pentenyl-O2 radicals

The rate coefficients for unimolecular reactions of 1-pentenyl-O_2 radicals (P1) were calculated in this work (see Table S7.1), where two main routes are accessible with similar contribution: ring closure forming a 6-membered ring ($k \sim 1.6\times10^{-1}$ s^{-1}), and allylic 1,5-H-migration ($k \sim 1.2\times10^{-1}$ s^{-1}).

The cycloperoxide alkyl product formed from P1 will add O_2, (see Vereecken et al.[7]), forming peroxy radical P9. The study by Vereecken et al.[7] showed no fast unimolecular loss processes for P9, while reactions with RO_2 and HO_2 are modelled to contribute a loss of ~0.2 s^{-1}, which is also the dominant loss in the theoretically predicted and modelled loss processes. The observed time evolution for P9, however, cannot be reproduced unless a total loss process of ~1 s^{-1} is included in the mechanism. The expected chemistry also does not show formation pathways to acrolein and C_2H_3OOH in yields that are sufficient to explain the peak heights observed in the mass spectra, nor to formation of CH_3O_2 and a C4H6O compound tentatively assigned to a methacrolein (CH_2=C(CH_3)-CH=O) or isomers (e.g. CH_3-CH=CH-CHO, CH_2=CH-C(=O)-CH_3, or CH_2=CH-CH_2-CH=O). We refer to the discussion in section S6 regarding fragmentation of the c-QO2 P9 intermediate as a source of these hydroperoxides and unsaturated aldehydes.

The allyl-1,5-H-migration leads to the resonance-stabilized P5 hydroperoxide-allyl radical. For such resonance-stabilized radicals it is known that O_2 addition is slower than for alkyl radicals, and reversible.[16, 24] The O_2 adducts P8 and P17 are therefore re-equilibrating continuously. The rate at which this happens is unknown, but for isoprene-derived hydroxy-allyl-radicals it was determined that this happens at an order of magnitude of 1 s^{-1} for isomers where the H-bonding group is not adjacent to the added OO (where we assume OH and OOH have a similar impact).[16, 24] A second effect playing is that the H-atom of the –OOH group is readily exchanged with the –OO$^{\bullet}$ radical group, where this so-called H-scrambling occurs at rates of the order of 10^2 s^{-1}.[5] The net result of these interconversions is that isomers P8a, P8b, P17a and P17b (and allyl radical P5 in much lower concentrations) are re-equilibrating at a timescale of about 1 s. As discussed extensively by Vereecken and Nozière[5], this pool of RO_2 radicals then typically disappears through the fastest loss channel accessible to all isomers. In this case this is the cyclisation reactions of P8b to a cyclic peroxide P20, where the elementary reaction is predicted by the theoretical work of Vereecken et al.[7] to occur at a rate close to 50 s^{-1}. Accounting for the pooling across the four RO_2 intermediates, this would lead of an effective loss rate of ~12 s^{-1} if the RO_2 were re-equilibrated instantly, but is probably closer to k~1-5 s^{-1} overall when accounting for the somewhat slower re-equilibration by O_2-elimination-readdition via allyl radical P5. The slower isomerization through P5 also allows cyclisation in P17b to have a minor contribution (elementary reaction rate ~ 1.6 s^{-1}, Vereecken et al.[7]) for that fraction of P5 where the initial O_2 addition leads to P17. The effective lifetime in the order of a second estimated thus is in agreement with the experimental observation, where little to no P12/P14 isomers were observed.

The observations also found intense signals at masses corresponding to C_5H_8O-H^+ and C_5H_8O-H_3O^+ clusters. Possible formation pathways are discussed in detail in section S6, and some of these are shown in scheme S6.14 shown above. Though this analysis is highly tentative at best, we attempted to probe some pathways. Observing the evolution of some ion signals as NO (> 10 ppm) is periodically added into the sampling line of the CIMS (Figure S6.15) can help in this analysis. As explained in the Experimental Section, excess NO is added periodically in the sampling line (0.2 s of residence time, Pressure ~ 0.1 atm) to distinguish the signals from RO_2 radicals from those of other compounds. The signals systematically decreasing when NO is added are thus those of peroxy radicals while those increasing are those of their reaction products with NO formed in the sampling line (i.e. produced in addition to the stable products formed in the reactor). The RO_2 P1 and CH_3O_2 were the only peroxy radicals found to be produced in the 1-pentenyl-O_2 system. Fig. S6.15 shows that the signals for these radicals indeed decrease when NO is added, while the signal at *m/z* 85 (C_5H_8O-H^+) is not affected by NO and a small fraction of the signal at *m/z* 103 increases systematically. The fraction of the signal at *m/z* 103 that increases with NO corresponds to the main product of the reaction of the peroxy radical P1 with NO and, more specifically, of the alkoxy radical P4. As explained in Section S6, in Mechanism C (cyclisation of unsaturated alkoxy radical followed by O_2 addition and HO_2 elimination) the 5-membered P10c isomer is expected to be the main product of the alkoxy radical P4 Rate coefficients were calculated theoretically (Table S7.2) for H-migration and ring closure in this pathway, while HCHO elimination was estimated from the SAR by Vereecken and Peeters.[9] Other isomers of P10 can, however, be produced by other pathways, such as from the linear alkyl radical P5 (cf. Scheme S10). The fraction of the *m/z* 103 signal increasing when adding NO corresponds to the main product of the alkoxy radical P4, i.e. is predominantly the cyclic product P10c. The large fraction of the signal at *m/z* 103 present in the absence of NO and the fact that the signal at *m/z* 85 is unaffected by NO suggests that these signals result from multiple isomers of P10, produced by other pathways. Note that, while *m/z* 103 also corresponds to the carbonyl product P3 produced directly by RO_2 + RO_2 (C_5H_8O-H_3O^+) or even to the hydroperoxide from the reaction RO_2+HO_2 ($C_5H_{10}O_2$-H^+) both compounds would be suppressed by the addition of NO.

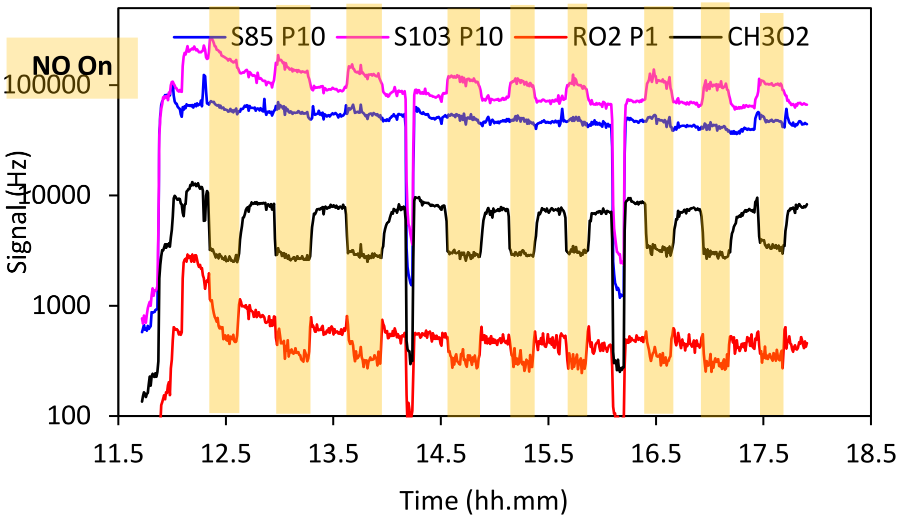

Figure S6.15: Dependence of the 1-pentenyl-O_2 and cycloether signals on addition of NO in the sampling line. Times where the RO2 signal goes down correspond to periods with added NO. The Mass 85 signal is barely affected, while the mass 103 signal goes up with NO.

In the reaction chamber, the formation of C5H8O through alkoxy radical chemistry would primarily be driven by the RO product channel of RO_2+RO_2 mutual reactions. This would be mainly by alkoxy radicals from P1, with some contributions from P17, where P8 cyclizes quickly and will be present in negligible concentrations. Our current rate coefficients in the kinetic model, however, suggest that these bimolecular reactions may not be important enough to explain the strength of the observed signals. Direct formation of P10 from P5 (“mechanism B”) seems unlikely due the high energy barrier and the low concentration of P5. The impact of protonated mechanisms (“mechanism F”) cannot be reliably estimated and is purely speculative.

1-hexenyl-O_2 annotated mechanism

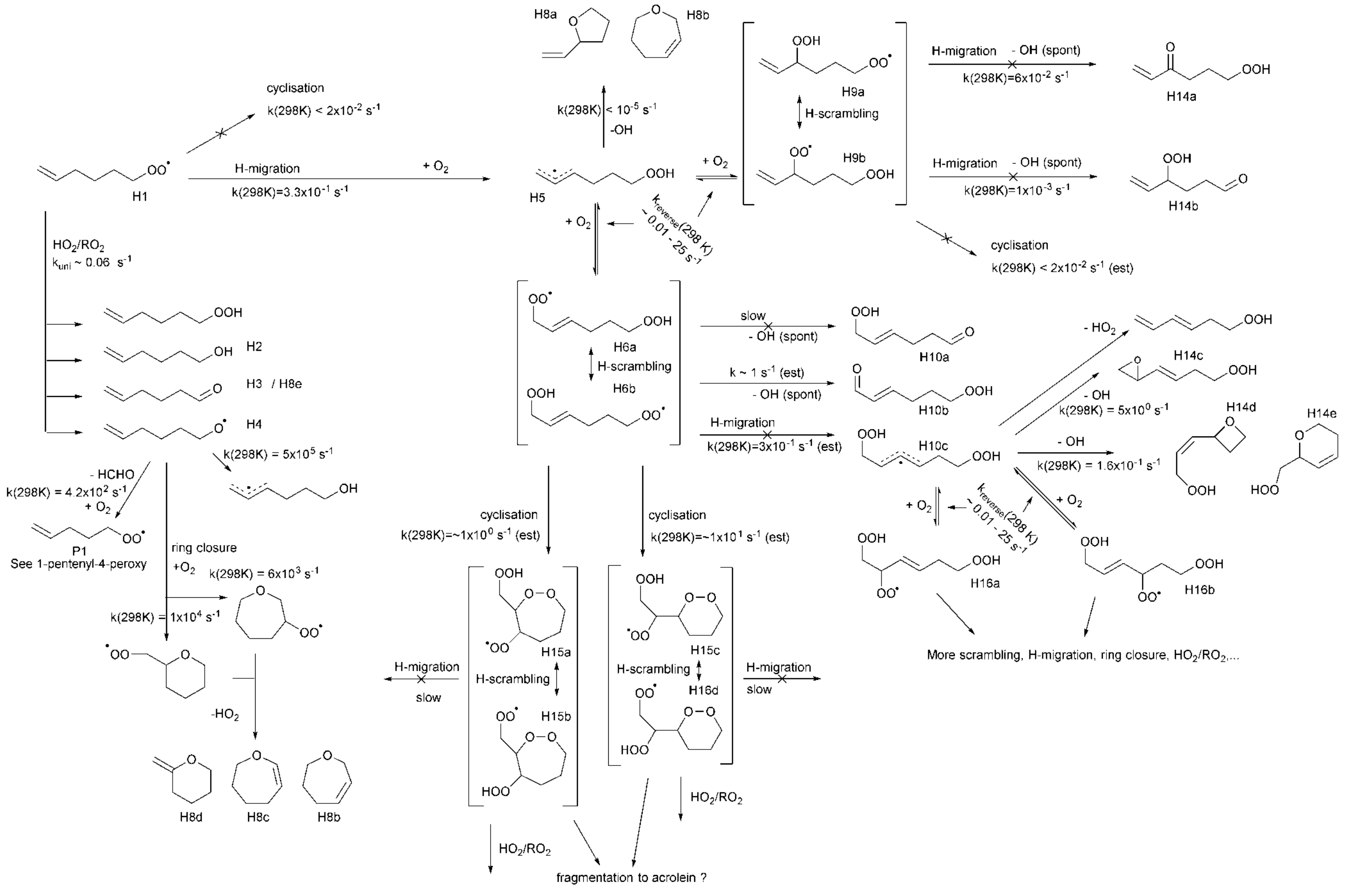

Figure S6.16: Extended mechanism for the oxidation of 1-hexenyl-O_2 radicals.

The rate coefficients for unimolecular reactions of 1-hexenyl-O_2 radicals (H1) were calculated in this work (see Table S7.1), where allylic 1,6-H-migration ($k \sim 3.3\times10^{-1}$ s^{-1}) was found to be the dominant loss process. The subsequent chemistry is largely analogous to that following the allyl-1,5-H-migration in 1-pentenyl-O_2. Due to the reversible O_2 addition on the hydroperoxide allylic radical H5, and the rapid H-scrambling in the resulting HOOQO_2 radicals (H6a, H6b, H9a, H9b), it is expected that these HOOQO_2 radicals equilibrate on a time scale of about a second. The H-migration processes in these radicals are estimated from the SARs by Vereecken and Nozière[5], where the rate of H-migration of an α-OOH-substituted, allylic H-atoms is tentatively estimated by combining the impact of each of these separate functionalities on the reaction rate as derived from those SARs. The rate of cyclisation of the unsaturated RO_2 is based on Vereecken et al. [7]. Most of the H-migrations are found to be slower than the ring closure reaction. The allylic dihydroperoxide H10c, although remaining a minor channel, has an interesting chemistry showing reversible O_2 addition and scrambling (similar to H5 and P5) as well as several pathways to epoxides, cyclic ethers, and double-unsaturated hydroperoxides. The provided rate coefficients for OH loss are estimated from Møller et al. [15] and Curran et al.[25]. The migration of the allylic α-OOH H-atom in H6b forming H10b is potentially a fast reaction, but likely remains slower than the cyclisation process. Overall, the main loss process for the pool of H6a, H6b, H9a, H9b, and H5 intermediates is ring closure to H15 for which 4 isomers can be formed. The work by Vereecken et al. (2021)[7] on H-migration in cyclic peroxide-peroxy radicals suggests that H-migration of ring-bound H-atoms is likely slow, while the 1,5-H-migrations between substituents are predicted to be non-competitive.[5] As such, the fate of the H15 product, HOO-cQO2, is likely determined by HO_2 and RO_2 reactions. Similar to the other cyclisation reaction of unsaturated RO_2 studied in this work, we observe a quantity of acrolein, which we assign to the decomposition of the cycloperoxide moiety. We refer to section S6 for an in-depth discussion on this fragmentation.

Observing the evolution of the main product signals, at *m/z* 99 and 117, corresponding to $C_6H_{10}O$-H+ and $C_6H_{10}O$-H_3O^+, respectively, and at m/z 81 attributed to their dehydrated ion as NO is periodically added in the sampling line (Fig. S6.2) provides additional information on the mechanisms. Possible formation pathways for these products are discussed in detail in section S6.2 and some are shown in Fig. S6.16 above. As in the analysis of the 1-pentenyl-O_2 system we attempted to validate "Mechanism C" for the formation of these $C_6H_{10}O$ products by observing their variation upon addition of NO in the sampling line (Fig. S6.16). Fig. S6.17 shows that, unlike in the 1-pentenyl-O_2 system, the signals for these products are unaffected by shifting the H1 chemistry towards the H4 alkoxy radical, although this channel is expected to produce isomers of the cyclic $C_6H_{10}O$ products (H8b/c/d). One possible explanation for this is that the amount for H8a/b produced in the absence of NO is exactly compensated by the amount of H8b/c/d produced in the presence of NO, thereby merely shifting the $C_6H_{10}O$ isomeric distribution without affecting the total concentration. Another, more likely explanation is, however, that the H4 alkoxy radical is not reacting through the cyclisation channel but undergoes an allyl-1,5-H-migration, not forming $C_6H_{10}O$ products and hence does not increasing its concentration in the sampling line. The rate coefficients shown in Fig. S6.16 are estimated by scaling the theoretically calculated 1-pentene-O_2 rate coefficients for ring closure and H-migration by a factor derived from the impact of the change in span[5, 7] on the rate coefficients of RO_2 ring closure (slowdown by about an order of magnitude and endocyclic allylic H-migration (acceleration by about 5 orders of magnitude). The rate of HCHO remains unchanged compared to P4.[9] This estimate favors allylic H-migration by over an order of magnitude, making additional $C_6H_{10}O$ formation in the sampling line under the influence of NO negligible; the measured $C_6H_{10}O$ remains unchanged from that present in the reaction chamber. We interpret this as further corroboration of "mechanism C". In the reaction chamber, formation of $C_6H_{10}O$ is unlikely to come through this mechanism starting at P4, however, as the pseudo-first order rate coefficient for bimolecular loss for P1 by HO_2/RO_2 is modelled to be slow compared to the allylic H-migration rate.

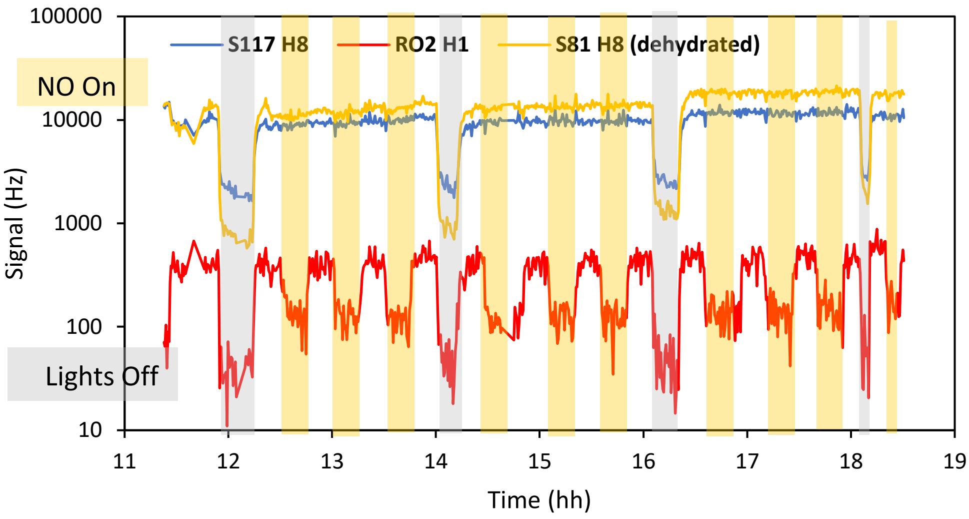

Figure S6.17: Dependence of the 1-hexenyl-O_2 and cycloether signals on addition of NO in the sampling line. Times where the RO2 signal goes down correspond to periods with added NO. Neither the 81 nor 117 mass signals seem affected by the NO addition.

2-Me-2-pentenyl-O2 annotated mechanism

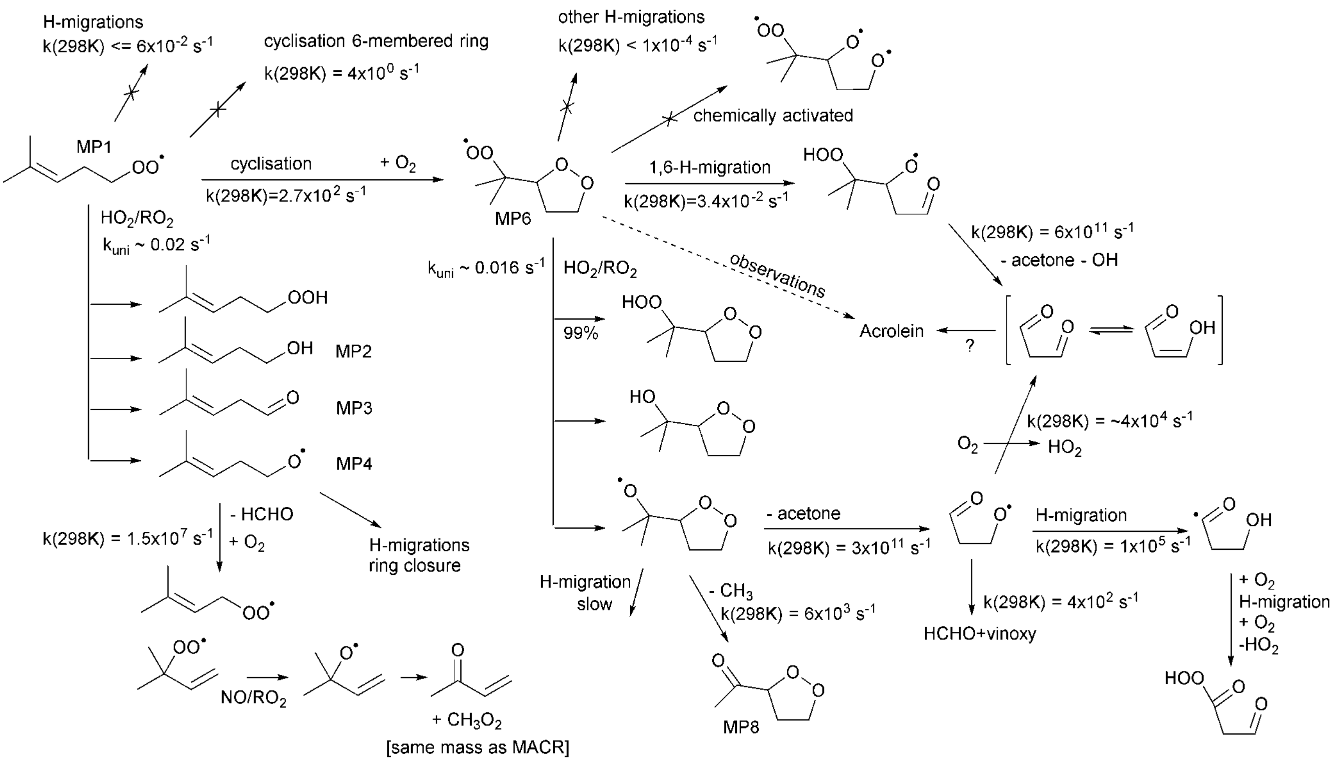

Figure S6.18: Extended mechanism for the oxidation of 2-Me-2-pentenyl-O2 radicals

The rate coefficients for unimolecular reactions of 2-methyl-2-pentenyl-O_2 radicals (MP1) were calculated in this work (see Table S7.1), where the dominant route is cyclisation to a 5-membered peroxide ring. Model calculations estimate the bimolecular loss for the RO_2 by reactions with HO_2/RO_2 at a pseudo-first order rate coefficient of about 2.5×10^{-3} s^{-1}, negligible compared to the unimolecular rate. The cycloperoxide alkyl product formed from MP1 will add O_2,[7] forming peroxy radical MP6. The study by Vereecken et al.[7] showed no fast unimolecular loss processes for B8, while reactions with RO_2 and HO_2 are modelled to contribute a loss of ~0.24 s^{-1}, which is also the dominant loss in the theoretically predicted and modelled loss processes. The observed time evolution for MP6, however, cannot be reproduced unless an additional unimolecular loss process of 10^6 to 10^7 $s^{-1}$is added to the mechanism. The expected chemistry also does not show formation pathways to acrolein. We refer to the discussion in section S6 regarding acrolein formation.

Section S7. Rate coefficients from theoretical calculations

The quantum chemical data for these calculations are available as a textfile in the repository at URL https://doi.org/10.26165/JUELICH-DATA/ZGIZV3 . This repository contains geometries, rotational constant, vibrational wavenumbers, and energies at various levels of theory.

Table S7.1

Theoretically calculated rate coefficients based on CCSD(T)/aug-cc-pVTZ//M06-2X-D3 quantum chemical data. Shown are the barrier height (E_b, kcal mol^{-1}), rate coefficients at 298 K (s^{-1}), and the parameters for the temperature-dependent rate coefficient between 200 and 450 K given as $k(T) = A \times (T/K)^n \times \exp(-E_a/T)$ with A in s^{-1} and E_a in K.

Reactant	Reaction	E_b	k(298 K)	A	n	E_a
CH_2=CH-CH_2-CH_2-OO$^•$	5-membered ring closure	14.1	5.3×10^{0}	2.14E+05	1.68	6013
	6-membered ring closure	16.8	3.1×10^{-2}	1.27E+07	0.96	7547
	Allylic 1,4-H-migration	29.4	1.9×10^{-5}	1.04E-90	31.87	-4397
	Butadiene + HO_2	29.2	5.8×10^{-10}	1.79E-27	12.76	9661
CH_2=CH-CH_2-CH_2-CH_2-OO$^•$	6-membered ring closure	16.2	1.6×10^{-1}	2.25E+03	2.18	6559
	7-membered ring closure	18.4	4.7×10^{-3}	2.47E+06	1.22	8060
	Allylic 1,5-H-migration	20.3	1.2×10^{-1}	2.29E-70	25.98	-3050
CH_2=CH-CH_2-CH_2-CH_2-CH_2-OO$^•$	7-membered ring closure	17.9	2.3×10^{-3}	5.59E+04	1.62	7811
	8-membered ring closure	16.3	1.0×10^{-2}	1.55E+02	2.32	6807
	1,5-H-migration	22.5	4.3×10^{-4}	5.50E-14	7.83	6500
	Allylic 1,6-H-migration	18.8	3.3×10^{-1}	2.66E-62	23.07	-2747
$(CH_3)_2$C=CH-CH_2-CH_2-OO$^•$	5-membered ring closure	12.2	2.7×10^{2}	6.11E+152	-49.57	18853
	6-membered ring closure	13.8	3.8×10^{0}	1.92E+08	0.48	6106
	Allylic 1,4-H-migration	28.0	9.4×10^{-5}	1.58E-91	32.31	-4690
	Allylic 1,7-H-migration (syn-CH_3)	20.0	6.1×10^{-2}	3.54E-58	21.54	-2022
	Allylic 1,7-H-migration (anti-CH_3)	34.3	6.3×10^{-10}	6.30E-105	35.80	-4417
	4-methylpentadiene + HO_2	29.6	7.3×10^{-10}	1.04E-26	12.53	9719
C$^•$$H_2$-$CH_2$-$CH_2$-$CH_2$OOH	Cyclic ether + OH (5-membered ring)	13.8	5.4×10^{1}	9.71E-05	4.79	4183
CH_2=CH-C$^•$H-CH_2-CH_2OOH	Cyclic ether + OH (4-membered ring)	25.9	1.7×10^{-7}	5.69E-71	27.57	3299
	Cyclic ether + OH (6-membered ring)	28.3	8.9×10^{-10}	2.18E-36	15.29	7707
O=CH-CH_2-CH_2O$^•$	1,4-H-migration	10.4	1.2×10^{5}	1.03E-13	7.99	1172

Table S7.2.

Theoretically calculated rate coefficients based on M06-2X-D3/aug-cc-pVTZ quantum chemical data. Shown are the barrier height (E_b, kcal mol^{-1}), rate coefficients at 298 K (s^{-1}), and the parameters for the temperature-dependent rate coefficient between 200 and 450 K given as $k(T) = A \times (T/K)^n \times \exp(-E_a/T)$ with A in s^{-1} and E_a in K.

Reactant	Reaction	E_b	k(298 K)	A	n	E_a
$[CH_2{=}CH_2\text{-}C^{\bullet}H\text{-}CH_2\text{-}CH_2O^{\bullet}\text{—}H_2O]^+$	Cyclic ether + H_2O cation (4-membered ring)	3.4	1.1×10^{10}	6.50E+12	-0.1	172
$CH_2{=}CH\text{-}CH_2\text{-}CH_2\text{-}CH_2O^{\bullet}$	Allylic 1,4-H-migration	17.9	2.1×10^{0}	1.48E-59	22.70	-2049
	5-membered ring closure	7.9	4.4×10^{5}	6.34E+09	0.32	3398
	6-membered ring closure	8.7	6.2×10^{4}	3.16E+11	-0.39	3943
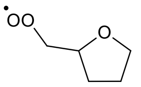	Concerted HO_2 elimination	37.1	1.4×10^{-15}	6.59E-46	18.85	11203
	1,4-H-migration	28.2	1.2×10^{-6}	6.18E-85	30.67	-1639
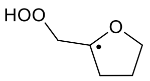	Unsaturated cyclic ether + HO_2	14.3	4.7×10^{1}	2.60E+07	1.74	6891
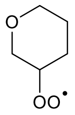	Concerted HO_2 elimination (β-OR side)	35.3	9.6×10^{-14}	5.69E-42	17.79	10850
	Concerted HO_2 elimination (alkyl side)	34.4	3.5×10^{-13}	4.62E-17	9.54	13537
	1,4-H-migration (β-OR side)	31.1	2.2×10^{-8}	5.65E-83	30.17	29
	1,4-H-migration (alkyl side)	35.1	1.4×10^{-10}	4.19E-69	25.24	2701
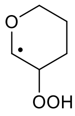	Unsaturated cyclic ether + HO_2	15.3	1.1×10^{2}	1.83E+12	0.37	7620
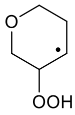	Unsaturated cyclic ether + HO_2	16.5	1.3×10^{0}	3.04E-03	5.10	6870
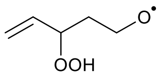	Cyclisation (6-membered ring)	10.2	3.7×10^{4}	1.32E+18	-2.46	5115
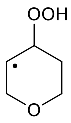	Unsaturated cyclic ether + HO_2	17.3	1.5×10^{0}	6.45E+06	2.10	8106
	Epoxidized cyclic ether + OH	15.4	1.8×10^{1}	2.99E-08	6.51	5041

Section S8. References

1. Keller-Rudek, H.; Moortgat, G.; Sander, R.; Sörensen, R., The mpi-mainz uv/vis spectral atlas of gaseous molecules of atmospheric interest. *Earth System Science Data* **2013,** *5*, 365-373.
2. Atkinson, R.; Baulch, D.; Cox, R.; Crowley, J.; Hampson, R.; Hynes, R.; Jenkin, M.; Rossi, M.; Troe, J.; Subcommittee, I., Evaluated kinetic and photochemical data for atmospheric chemistry: Volume ii–gas phase reactions of organic species. *Atmos. Chem. Phys.* **2006,** *6*, 3625-4055.
3. Atkinson, R.; Baulch, D.; Cox, R. A.; Crowley, J.; Hampson, R.; Hynes, R.; Jenkin, M.; Rossi, M.; Troe, J., Evaluated kinetic and photochemical data for atmospheric chemistry: Volume i-gas phase reactions of o x, ho x, no x and so x species. *Atmos. Chem. Phys.* **2004,** *4*, 1461-1738.
4. Jenkin, M. E.; Valorso, R.; Aumont, B.; Rickard, A. R., Estimation of rate coefficients and branching ratios for reactions of organic peroxy radicals for use in automated mechanism construction. *Atmos. Chem. Phys.* **2019,** *19*, 7691-7717.
5. Vereecken, L.; Nozière, B., H migration in peroxy radicals under atmospheric conditions. *Atmos. Chem. Phys.* **2020,** *20*, 7429-7458.
6. Atkinson, R.; Baulch, D.; Cox, R.; Crowley, J.; Hampson, R.; Hynes, R.; Jenkin, M.; Rossi, M.; Troe, J., Evaluated kinetic and photochemical data for atmospheric chemistry: Volume iii–gas phase reactions of inorganic halogens. *Atmos. Chem. Phys.* **2007,** *7*, 981-1191.
7. Vereecken, L.; Vu, G.; Wahner, A.; Kiendler-Scharr, A.; Nguyen, H., A structure activity relationship for ring closure reactions in unsaturated alkylperoxy radicals. *Phys. Chem. Chem. Phys.* **2021,** *23*, 16564-16576.
8. Burkholder, J.; Sander, S.; Abbatt, J.; Barker, J.; Cappa, C.; Crounse, J.; Dibble, T.; Huie, R.; Kolb, C.; Kurylo, M. *Chemical kinetics and photochemical data for use in atmospheric studies; evaluation number 19*; Pasadena, CA: Jet Propulsion Laboratory, National Aeronautics and Space …: 2020.
9. Vereecken, L.; Peeters, J., Decomposition of substituted alkoxy radicals—part i: A generalized structure–activity relationship for reaction barrier heights. *Phys. Chem. Chem. Phys.* **2009,** *11*, 9062-9074.
10. Novelli, A.; Cho, C.; Fuchs, H.; Hofzumahaus, A.; Rohrer, F.; Tillmann, R.; Kiendler-Scharr, A.; Wahner, A.; Vereecken, L., Experimental and theoretical study on the impact of a nitrate group on the chemistry of alkoxy radicals. *Phys. Chem. Chem. Phys.* **2021,** *23*, 5474-5495.
11. Vereecken, L.; Nguyen, T. L.; Hermans, I.; Peeters, J., Computational study of the stability of α-hydroperoxyl- or α-alkylperoxyl substituted alkyl radicals. *Chem. Phys. Lett.* **2004,** *393*, 432-436.
12. Villano, S. M.; Huynh, L. K.; Carstensen, H.-H.; Dean, A. M., High-pressure rate rules for alkyl+ o2 reactions. 2. The isomerization, cyclic ether formation, and β-scission reactions of hydroperoxy alkyl radicals. *J. Phys. Chem. A* **2012,** *116*, 5068-5089.
13. Bugler, J.; Power, J.; Curran, H. J., A theoretical study of cyclic ether formation reactions. *Proceedings of the Combustion Institute* **2017,** *36*, 161-167.
14. Miyoshi, A., Systematic computational study on the unimolecular reactions of alkylperoxy (ro2), hydroperoxyalkyl (qooh), and hydroperoxyalkylperoxy (o2qooh) radicals. *J. Phys. Chem. A* **2011,** *115*, 3301-3325.
15. Møller, K. H.; Kurtén, T.; Bates, K. H.; Thornton, J. A.; Kjaergaard, H. G., Thermalized epoxide formation in the atmosphere. *J. Phys. Chem. A* **2019,** *123*, 10620-10630.
16. Novelli, A.; Vereecken, L.; Bohn, B.; Dorn, H.-P.; Gkatzelis, G. I.; Hofzumahaus, A.; Holland, F.; Reimer, D.; Rohrer, F.; Rosanka, S., Importance of isomerization reactions for oh radical regeneration from the photo-oxidation of isoprene investigated in the atmospheric simulation chamber saphir. *Atmos. Chem. Phys.* **2020,** *20*, 3333-3355.
17. Goldman, M. J.; Green, W. H.; Kroll, J. H., Chemistry of simple organic peroxy radicals under atmospheric through combustion conditions: Role of temperature, pressure, and no x level. *J. Phys. Chem. A* **2021,** *125*, 10303-10314.

18. Rienstra-Kiracofe, J. C.; Allen, W. D.; Schaefer, H. F., The c2h5+ o2 reaction mechanism: High-level ab initio characterizations. *J. Phys. Chem. A* **2000,** *104*, 9823-9840.

19. Orlando, J. J.; Tyndall, G. S.; Wallington, T. J., The atmospheric chemistry of alkoxy radicals. *Chem. Rev.* **2003,** *103*, 4657-4690.

20. Davis, A. C.; Francisco, J. S., Ab initio study of hydrogen migration in 1-alkylperoxy radicals. *J. Phys. Chem. A* **2010,** *114*, 11492-11505.

21. Davis, A. C.; Francisco, J. S., Ab initio study of hydrogen migration across n-alkyl radicals. *J. Phys. Chem. A* **2011,** *115*, 2966-2977.

22. Nozière, B.; Vereecken, L., Direct observation of aliphatic peroxy radical autoxidation and water effects: An experimental and theoretical study. *Angew. Chem. Int. Ed.* **2019,** *58*, 13976-13982.

23. Winkelman, J.; Voorwinde, O.; Ottens, M.; Beenackers, A.; Janssen, L., Kinetics and chemical equilibrium of the hydration of formaldehyde. *Chem. Eng. Sci.* **2002,** *57*, 4067-4076.

24. Medeiros, D. J.; Blitz, M. A.; Seakins, P. W.; Whalley, L. K., Direct measurements of isoprene autoxidation: Pinpointing atmospheric oxidation in tropical forests. *JACS Au* **2022,** *2*, 809-818.

25. Curran, H. J.; Gaffuri, P.; Pitz, W. J.; Westbrook, C. K., A comprehensive modeling study of n-heptane oxidation. *Combust. Flame* **1998,** *114*, 149-177.
